# The effects of stress on eyewitness memory: A survey of memory experts and laypeople

**DOI:** 10.3758/s13421-020-01115-4

**Published:** 2020-11-25

**Authors:** Carey Marr, Henry Otgaar, Melanie Sauerland, Conny W. E. M. Quaedflieg, Lorraine Hope

**Affiliations:** 1 Faculty of Psychology and Neuroscience, Maastricht University, Universiteitssingel 40, Maastricht, The Netherlands 6229 ER; 2 Department of Psychology, University of Portsmouth, Portsmouth, UK; 3 Faculty of Law, Catholic University of Leuven, Leuven, Belgium

**Keywords:** Stress, Memory, Expert, Laypeople, Commonsense belief

## Abstract

This survey examined lay and expert beliefs about statements concerning stress effects on (eyewitness) memory. Thirty-seven eyewitness memory experts, 36 fundamental memory experts, and 109 laypeople endorsed, opposed, or selected *don’t know* responses for a range of statements relating to the effects of stress at encoding and retrieval. We examined proportions in each group and differences between groups (eyewitness memory experts vs. fundamental memory experts; experts vs. laypeople) for endorsements (*agree* vs. *disagree*) and selections (*don’t know* vs. *agree/disagree*). High proportions of experts from both research fields agreed that very high levels of stress impair the accuracy of eyewitness testimony. A majority of fundamental experts, but not eyewitness experts, endorsed the idea that stress experienced during encoding can enhance memory. Responses to statements regarding moderating factors such as stressor severity and detail type provided further insight into this discrepancy. Eyewitness memory experts more frequently selected the *don’t know* option for neuroscientific statements regarding stress effects on memory than fundamental memory experts, although *don’t know* selections were substantial among both expert groups. Laypeople’s responses to eight of the statements differed statistically from expert answers on topics such as memory in *children*, in *professionals* such as police officers, for *faces* and *short crimes*, and the existence of *repression*, providing insight into possible ‘commonsense’ beliefs on stress effects on memory. Our findings capture the current state of knowledge about stress effects on memory as reflected by sample of experts and laypeople, and highlight areas where further research and consensus would be valuable.

**Supplementary Information:**

The online version contains supplementary material available at 10.3758/s13421-020-01115-4.

Witnesses often experience acute stress in forensic contexts, whether during a crime or during subsequent police interviews (Bornstein, Hullman, & Miller, 2013; J. A. Davis, 2016; Yuille & Cutshall, 1986). Facing difficult, frightening, and emotional events can trigger a subjective and physiological stress response from the witness (Bornstein & Robicheaux, 2009). A body of research has been devoted to examining the potential effects of acute stress on memory, but results have been inconsistent. Equally important, there is little information about current memory experts’ and laypeople’s knowledge about the stress–memory relationship. Memory experts in different research domains, such as eyewitness memory experts and fundamental memory experts, as well as laypeople, may have different understandings of this relationship. If different perspectives exist, such differences could emerge in courtroom settings in problematic ways. For example, memory researchers from different fields might be asked to be expert witnesses in court, and then provide diverging statements concerning stress–memory relationships. Additionally, laypeople acting as jurors may evaluate eyewitness evidence based on their preexisting ‘commonsense’ beliefs. To capture the contemporary perspectives of memory experts and laypeople, we examined current memory experts’ and laypeople’s beliefs about the effects of acute stress on memory by means of a targeted survey.

## Stress and memory: An ongoing discussion

Two groups of memory researchers have examined the effects of acute stress on memory encoding and retrieval. One group predominantly concentrates on memory in applied settings, such as eyewitness memory, and the other group mainly focuses on fundamental memory research, including neurobiological research related to basic memory processes (e.g., memory performance for noncomplex stimuli, such as word lists or numeric strings). Generally, across fields, research shows that acute stress at *retrieval* impairs memory (e.g., Schwabe, Joëls, Roozendaal, Wolf, & Oitzl, 2012; Shields, Sazma, McCullough, & Yonelinas, 2017; Wolf, 2017), although limited research on this specific issue has been conducted in the eyewitness memory context (see Dellapaolera, 2019; Robicheaux, 2016). However, findings concerning the effects of acute stress at *encoding* on memory performance appear to be discrepant between research fields (Christianson, 1992; Schwabe et al., 2012; Shields et al., 2017). Eyewitness memory research mostly concludes that encoding stress impairs eyewitness memory. For example, a meta-analysis of 27 eyewitness memory studies suggested that heightened stress exerts a negative effect on eyewitness memory for both the perpetrator and details associated with the crime (Deffenbacher, Bornstein, Penrod, & McGorty, 2004). Eyewitness memory researchers often cite such research as evidence that the negative effect of encoding stress on memory is a conclusive finding (e.g., Schmechel, O’Toole, Easterly, & Loftus, 2006; Yarmey & Jones, 1983). For example, Schmechel et al. (2006) stated that “highly stressful situations may make an experience seem especially vivid, but such stressors can reduce the ability to recall details about a person’s face”, declaring this summary of stress effects as an “empirical answer” (p. 179). Field studies in this area have also highlighted a negative effect of severe encoding stress on memory (e.g., Metcalfe, Brezler, McNamara, Malette, & Vuorre, 2019; Stanny & Johnson, 2000; Valentine & Mesout, 2008). For instance, in one study, active-duty military personnel participated in a survival school training exercise (Morgan et al., 2004). During training, participants experienced one low-stress interrogation and one high-stress interrogation and were later asked either to make identification decisions for each of the two interrogations from a live lineup (Study 1) or a photo lineup (Study 2). In two subsequent studies, all participants were either in the high stress (Study 3) or low stress condition (Study 4) and made an identification decision from a sequential photo lineup. Regardless of assessment method, identification performance was better for low-stress interrogators compared with high-stress interrogators. However, it should be noted that several other factors in this field study are potentially confounding variables, such as the fact that all soldiers participating in the research were deprived of food and sleep for 48 hours prior to the interrogations. These naturalistic elements of the survival training context likely impacted the stress–memory relationship beyond the effects of acute stress alone.

The view that stress at encoding negatively affects subsequent memory is in contrast to findings reported in fundamental memory research, which demonstrate that acute stress at encoding can actually enhance memory performance (e.g., Henckens, Hermans, Pu, Joëls, & Fernández, 2009; Shields et al., 2017; Vogel & Schwabe, 2016; Wolf, 2012). These findings can be accounted for in terms of the cognitive effects of physiological stress responses triggered by acute stress. When we experience acute stress, adrenaline and noradrenaline are quickly released, followed by the slower release of cortisol from the activation of the hypothalamic–pituitary–adrenal (HPA) axis (e.g., Joëls & Baram, 2009; Joëls, Fernández, & Roozendaal, 2011; Robbins, 1984; Ulrich-Lai & Herman, 2009). The rapid catecholaminergic and nongenomic glucocorticoid actions set the brain in a memory formation mode (Diamond, Campbell, Park, Halonen, & Zoladz, 2007; Joëls, Pu, Wiegert, Oitzl, & Krugers, 2006). If encoding occurs during this part of the memory phase, acute stress should enhance memory formation for stress-related material, while also impairing retrieval of material unrelated to the stressor (e.g., Diamond et al., 2007; Joëls et al., 2006; Quaedflieg & Schwabe, 2018; Shields et al., 2017). Methodological differences between eyewitness research and fundamental memory research may explain these contradictory results. For example, differences in the type and severity of stressors, the timing between a stressor and encoding, and retention intervals between encoding and retrieval could result in varied findings (for discussions of potential participant and study design moderators, see Sauerland et al., 2016; Shields, 2020; Shields et al., 2017; Thomas & Karanian, 2019).

These diverging research findings with respect to how stress at encoding impacts memory performance suggest that disagreement might also exist between different types of experts about topics related to acute stress and memory. Additionally, beliefs held by the general population about stress and memory do not always mirror expert knowledge (e.g., Yarmey & Jones, 1983). Past surveys have examined some general beliefs about stress and memory among both lay and expert samples. Table 1 presents an overview of 17 published surveys that we located on this topic, published from 1979, with the most recent published in 2010. Across all 17 surveys, 79% of laypeople agreed that high stress harms the accuracy of eyewitness testimony (survey responses ranging from 41% to 92%). Three surveys examining experts’ beliefs about the negative effects of stress on eyewitness memory between 1983 and 2001 show a slight decline in agreement (Kassin, Ellsworth, & Smith, 1989; Kassin, Tubb, Hosch, & Memon, 2001; Yarmey & Jones, 1983). In 1983, 88% of experts (*N* = 16) agreed with the statement that *When a person experienced extreme stress as the victim of a crime, he/she will have reduced ability to notice and remember the details of the event* (Yarmey & Jones, 1983)*.* In 1989, 73% of eyewitness experts (*N* = 63) agreed that the statement *Very high levels of stress impair the accuracy of eyewitness testimony* was reliable enough to present in court (Kassin et al., 1989). By 2001, agreement levels had dropped to 60% (*N* = 62; Kassin et al., 2001). Similarly, 79% of experts agreed that the evidence supported that statement in 1989, whereas 11 years later, 65% of experts agreed that high levels of stress impaired the accuracy of eyewitness testimony. These surveys among experts suggest that consensus concerning the stress–memory relationship has been declining over the years. However, the statement used in previous surveys does not include an indication of memory phase (i.e., encoding or retrieval). Additionally, the most recent survey investigating expert opinions on this relationship is nearly 2 decades old (Kassin et al., 2001), and many studies regarding stress and memory have been published since then. For example, all 90 papers included in the Shields et al. (2017) meta-analysis on this topic were published in or after 2001, highlighting the need for a more contemporary assessment of opinion.

**Table 1:** Percentage of layperson respondents agreeing with the statement on the negative effects of high stress on eyewitness memory in past surveys

Authors	Year	Country	Sample	% endorsed
Loftus	1979	USA	500 students	67
Yarmey & Jones	1983	Canada	60 students and 60 local adults	57
Deffenbacher & Loftus	1982	USA	76 students	85
Deffenbacher & Loftus	1982	USA	100 students	79
Deffenbacher & Loftus	1982	USA	46 jurors	41
Deffenbacher & Loftus	1982	USA	43 jurors	53
Noon & Hollin	1987	UK	28 students	79
Noon & Hollin	1987	UK	24 law students	79
Noon & Hollin	1987	UK	24 potential jurors	67
Kassin & Barndollar	1992	USA	39 students and 40 local adults	82
Schmechel, O’Toole, Easterly, & Loftus*	2004	USA	1,007 potential jurors	80
Benton, Ross, Bradshaw, Thomas, & Bradshaw	2006	USA	111 jurors	68
Read & Desmarais	2009	Canada	201 potential jurors	79
Read & Desmarais	2009	Canada	200 potential jurors	92
Read & Desmarais	2009	Canada	598 potential jurors	88
Magnussen, Melinder, Stridbeck, & Raja	2010	Norway	164 members of juror pool	79
Magnussen, Melinder, Stridbeck, & Raja	2010	Norway	1,000 potential jurors	84
			**4,321 participants**	**79.15** **(3,420 participants)**

*Note*. % endorsed = percentage of participants who believed in negative effects of high stress on eyewitness memory. Potential jurors = general public. * = Statement in this survey was “if an eyewitness was under high stress at the time of the crime, the eyewitness will have better recall for the details of the event”; percentage in table represents those who believed this statement was false

Other surveys focused on beliefs about emotional events. For example, in one survey, 80% of layperson respondents endorsed the notion that emotional events are usually remembered more accurately than memories for everyday events (Conway, Justice, & Morrison, 2014). More recently, 54% of a surveyed lay sample agreed or strongly agreed that experiences involving very strong emotions and memories of emotionally negative experiences were more accurately remembered than emotionally moderate or weak, neutral, or positive experiences (Akhtar, Kalin, Thurow, Rosenkranz, & Davidson, 2018). The finding that most laypeople believe that emotional intensity gives rise to accurate memories seems to be out of line with other surveys indicating that laypeople generally believe acute stress harms eyewitness memory. However, although emotional intensity and stress often relate to similar applied matters, the two cannot be fully equated. For example, eyewitness scenarios often involve both negative emotionality and stress (e.g., witnessing an unexpected fatal car accident or life-threatening assault). Other experiences, however, may be emotionally negative, but not necessarily elicit an acute stress response (e.g., a failed relationship or the death of an ill parent). The relationship between emotional intensity and memory accuracy has also been investigated in one expert sample (Akhtar et al., 2018). Forty-six percent of experts agreed or strongly agreed with the idea that emotional experiences were more accurately remembered than neutral or positive experiences. However, 54% of experts disagreed with this statement, suggesting a similar lack of consensus between experts regarding topics associated with the effects of acute stress on memory.

Past surveys investigating stress and memory have typically included a single statement concerning the effects of acute stress on memory (i.e., *Very high levels of stress impair the accuracy of eyewitness testimony*). However, the complexity of the effects of acute stress on memory cannot be meaningfully captured in this single item. A more in-depth investigation of laypeople’s and experts’ understandings about effects of stress on memory is valuable for two reasons. First, the complexity of this particular topic is evident through the numerous moderators about which beliefs have not yet been examined. Specifically, previous surveys have not included questions about specificity of stressor timing (i.e., encoding vs. retrieval; see Joëls et al., 2011; Quaedflieg & Schwabe, 2018), the neuroscientific theories behind stress effects on memory, and the potential moderators of the acute stress–memory relationship (i.e., age, type of memory test, stress severity, detail type, etc.). Understanding expert beliefs about these moderators will also elucidate which factors require further investigation, setting important directions for future research on this topic. Second, understandings about stress and memory can have real-life consequences. Laypeople’s views may enter the legal decision-making process when they act as jurors, and research suggests their beliefs can impact their decisions about credibility and guilt (Bornstein, O’Bryant, & Zickafoose, 2008). Furthermore, experts’ opinions can also affect legal decision-making when they testify as expert witnesses. Indeed, the effects of stress on the accuracy of eyewitness testimony was identified as the topic second most frequently testified about by experts across 21 eyewitness-related topics (Kassin et al., 1989). Therefore, even though the different research fields focusing on stress and memory do not show conclusive findings, understanding laypeople’s and expert’s beliefs with respect to this topic is still vital due to these potential real-world consequences.

## The current survey

The current survey assessed laypeople’s and experts’ beliefs about the relationship between acute stress and memory. The survey items used are presented in Table 2. We targeted a group of lay respondents and two groups of expert respondents, eyewitness memory researchers and fundamental memory researchers (i.e., those investigating basic memory processes). We examined beliefs using a variety of statements concerning the effects of stress on memory. The primary interest of this exploratory survey was to examine what experts from both fields and laypeople believe about these statements. We did not make explicit specific predictions for the survey about current laypeople’s and experts’ beliefs.

**Table 2:** Survey statements about stress and memory with percentage of participant endorsement

Statement	Shorthand	Percentage of participant endorsement			
			Eyewitness memory experts *n* = 37	Fundamental memory experts *n* = 36	Laypeople *n* = 109
1. Very high levels of stress impair the accuracy of eyewitness testimony.	*high stress impairs*	Agree	**94.6** (87.3, 100.0)	**80.6** (67.7, 93.5)	**93.6** (89.0, 98.2)
		Disagree	**2.7** (0.0, 7.9)	**16.7** (4.5, 28.9)	**3.7** (0.2, 7.2)
		Don’t know	**2.7** (0.0, 7.9)	**2.8** (0.0, 8.2)	**2.8** (0.0, 5.9)
2. If an eyewitness is stressed during a police interview (i.e., at retrieval), his or her memory will be less accurate than if he or she were not stressed.	*police interview*	Agree	**81.1** (68.5, 93.7)	**75.0** (60.9, 89.1)	**81.7** (74.4, 89.0)
		Disagree	**8.1** (0.0, 16.9)	**13.9** (2.6, 25.2)	**12.8** (6.5, 19.1)
		Don’t know	**10.8** (0.8, 20.8)	**11.1** (0.8, 21.4)	**5.5** (1.2, 9.8)
3. Experiencing stress while trying to remember something (i.e., at retrieval) impairs memory retrieval.	*stress impairs retrieval*	Agree	**86.5** (75.5, 97.5)	**91.7** (82.7, 100.0)	**78.0** (70.2, 85.8)
		Disagree	**2.7** (0.0, 7.9)	**8.3** (0.0, 17.3)	**12.8** (6.5, 19.1)
		Don’t know	**10.8** (0.8, 20.8)	**0.0**	**9.2** (3.8, 14.6)
4. Experiencing stress during an event (i.e., at encoding) enhances memory for that event.	*stress enhances encoding*	Agree	**32.4** (17.3, 47.5)	**77.8** (64.2, 91.4)	**33.9** (25.0, 42.8)
		Disagree	**62.2** (46.6, 77.8)	**19.4** (6.5, 32.2)	**53.2** (43.8, 62.6)
		Don’t know	**5.4** (0.0, 12.7)	**2.8** (0.0, 8.2)	**12.8** (6.5, 19.1)
5. Children’s memories are less affected by stress experienced during an event (i.e., at encoding) than adults’ memories.	*children less affected*	Agree	**5.4** (0.0, 12.7)	**2.8** (0.0, 8.2)	**27.5** (19.1, 35.9)
		Disagree	**73.0** (58.7, 87.3)	**55.6** (39.4, 71.8)	**56.9** (47.6, 66.2)
		Don’t know	**21.6** (8.3, 34.9)	**41.7** (25.6, 57.8)	**15.6** (8.8, 22.4)
6. Stress experienced during an event (i.e., at encoding) enhances memory for central details of the event, but not for peripheral details.	*detail type*	Agree	**78.4** (65.1, 91.7)	**80.6** (67.7, 93.5)	**45.0** (35.7, 54.3)
		Disagree	**10.8** (0.8, 20.8)	**16.7** (4.5, 28.9)	**35.8** (26.8, 44.8)
		Don’t know	**10.8** (0.8, 20.8)	**2.8** (0.0, 8.2)	**19.3** (11.9, 26.7)
7. When an eyewitness is stressed while trying to remember something (i.e., at retrieval), his or her free recall ability is more negatively affected by this stress than his or her recognition ability.	*test type*	Agree	**56.8** (40.8, 72.8)	**72.2** (57.6, 86.8)	**75.2** (67.1, 83.3)
		Disagree	**18.9** (6.3, 31.5)	**8.3** (0.0, 17.3)	**10.1** (4.4, 15.8)
		Don’t know	**24.3** (10.5, 38.1)	**19.4** (6.5, 32.3)	**14.7** (8.1, 21.3)
8. Stress affects memory for faces differently than memory for other types of stimuli.	*faces affected differently*	Agree	**37.8** (22.2, 53.4)	**19.4** (6.5, 32.3)	**64.2** (55.2, 73.2)
		Disagree	**32.4** (17.3, 47.5)	**38.9** (23.0, 54.8)	**11.9** (5.8, 18.0)
		Don’t know	**29.7** (15.0, 44.4)	**41.7** (25.6, 57.8)	**23.9** (15.9, 31.9)
9. The memory of trained professionals, such a police officers, will be less affected by stress than the memory of normal eyewitnesses.	*professionals less affected*	Agree	**13.5** (2.5, 24.5)	**19.4** (6.5, 32.3)	**64.2** (55.2, 73.2)
		Disagree	**86.5** (75.5, 97.5)	**72.2** (57.6, 86.8)	**29.4** (20.8, 38.0)
		Don’t know	**0.0**	**8.3** (0.0, 17.3)	**6.4** (1.8, 11.0)
10. A victim’s memory will typically be more affected by stress experienced during a crime (i.e., at encoding) than a bystander eyewitness’ memory.	*victims more affected*	Agree	**75.7** (61.9, 89.5)	**58.3** (42.2, 74.4)	**78.9** (71.2, 86.6)
		Disagree	**18.9** (6.3, 31.5)	**19.4** (6.5, 32.3)	**12.8** (6.5, 19.1)
		Don’t know	**5.4** (0.0, 12.7)	**22.2** (8.6, 35.8)	**8.3** (3.1, 13.5)
11. Eyewitnesses who experience stress during a crime are more likely to have memories that they unconsciously blocked due to trauma (i.e., “repressed memories”) than those who do not experience such stress.	*repression*	Agree	**16.2** (4.3, 28.1)	**13.9** (2.6, 25.2)	**85.3** (78.7, 91.9)
		Disagree	**75.7** (61.9, 89.5)	**69.4** (54.3, 84.5)	**7.3** (2.4, 12.2)
		Don’t know	**8.1** (0.0, 16.9)	**16.7** (4.5, 28.9)	**7.3** (2.4, 12.2)
12. Eyewitnesses have more difficulty remembering violent events than nonviolent ones.	*violent events*	Agree	**40.5** (24.7, 56.3)	**13.9** (2.6, 25.2)	**44.0** (34.7, 53.3)
		Disagree	**54.1** (38.0, 70.2)	**55.6** (39.4, 71.8)	**36.7** (27.7, 45.7)
		Don’t know	**5.4** (0.0, 12.7)	**30.6** (15.5, 45.7)	**19.3** (11.9, 26.7)
13. Stressful experiences that are emotional are generally better remembered than stressful experiences that are not emotional.	*emotional better remembered*	Agree	**62.2** (46.6, 77.8)	**61.1** (45.2, 77.0)	**52.3** (42.9, 61.7)
		Disagree	**18.9** (6.3, 31.5)	**22.2** (8.6, 35.8)	**29.4** (20.8, 38.0)
		Don’t know	**18.9** (6.3, 31.5)	**16.7** (4.5, 28.9)	**18.3** (11.0, 25.6)
14. Eyewitnesses who experience moderate levels of stress during a crime (i.e., at encoding) display better memory than eyewitnesses who experience low levels of stress during a crime.	*moderate stress*	Agree	**62.2** (46.6, 77.8)	**69.4 (**54.3, 84.5)	**35.8** (26.8, 44.8)
		Disagree	**18.9** (6.3, 31.5)	**16.7** (4.5, 28.9)	**50.5** (41.1, 59.9)
		Don’t know	**18.9** (6.3, 31.5)	**13.9** (2.6, 25.2)	**13.8** (7.3, 20.3)
15. Severe levels of stress, but not moderate levels of stress, generally harm eyewitness memory.	*severe stress*	Agree	**83.8** (71.9, 95.7)	**63.9** (48.2, 79.6)	**63.3** (54.3, 72.3)
		Disagree	**16.2** (4.3, 28.1)	**22.2** (8.6, 35.8)	**23.9** (15.9, 31.9)
		Don’t know	**0.0**	**13.9** (2.6, 25.2)	**12.8** (6.5, 19.1)
16. When an eyewitness experiences a relatively short crime (i.e., fewer than 5 minutes), his or her memories are not affected by this stress.	*short crime*	Agree	**2.7** (0.0, 7.9)	**0.0**	**26.6 (**18.3, 34.9)
		Disagree	**91.9** (83.1, 100.0)	**91.7** (82.7, 100.0)	**54.1** (44.7, 63.5)
		Don’t know	**5.4** (0.0, 12.7)	**8.3** (0.0, 17.3)	**19.3** (11.9, 26.7)
17. If one experiences stress during an event (i.e., at encoding), it is likely that his or her memories will be more abstract and general rather than specific and detailed.	*abstractness*	Agree	**37.8** (22.2, 53.4)	**41.7** (25.6, 57.8)	**57.8** (48.5, 67.1)
		Disagree	**35.1** (19.7, 50.5)	**52.8** (36.5, 69.1)	**24.8** (16.7, 32.9)
		Don’t know	**27.0** (12.7, 41.3)	**5.6** (0.0, 13.1)	**17.4** (10.3, 24.5)
18. If memory is immediately tested after a stressor, one does not experience a memory deficit; rather, memory at this stage can actually be enhanced.	*immediate retrieval enhances*	Agree	**29.7** (15.0, 44.4)	**22.2** (8.6, 35.8)	**46.8** (37.4, 56.2)
		Disagree	**37.8** (22.2, 53.4)	**41.7** (25.6, 57.8)	**31.2** (22.5, 39.9)
		Don’t know	**32.4** (17.3, 47.5)	**36.1** (20.4, 51.8)	**22.0** (14.2, 29.8)
19. Memory tested two hours after a stressor is experienced will be worse than memory tested 30 minutes after a stressor is experienced.	*retrieval timing*	Agree	**62.2** (46.6, 77.8)	**30.6** (15.5, 45.7)	**57.8** (48.5, 67.1)
		Disagree	**16.2** (4.3, 28.1)	**44.4** (28.2, 60.6)	**23.9** (15.9, 31.9)
		Don’t know	**21.6** (8.3, 34.9)	**25.0** (10.9, 39.1)	**18.3** (11.0, 25.6)
20. Stress that occurs before the presentation of incorrect information can protect an eyewitness’ original memory because stress prevents new information from being incorporated into existing memory.	*misinformation protection*	Agree	**10.8** (0.8, 20.8)	**19.4** (6.5, 32.3)	**45.9** (36.5, 55.3)
		Disagree	**43.2** (27.2, 59.2)	**69.4** (54.3, 84.5)	**30.3** (21.7, 38.9)
		Don’t know	**45.9** (29.8, 62.0)	**11.1** (0.8, 21.4)	**23.9** (15.9, 31.9)
21. Memories of older adults (ages 55+) are less affected by stress experienced during an event (i.e., at encoding) than memories of younger adults (ages 18–35).	*older adults less affected*	Agree	**5.4** (0.0, 12.7)	**8.3** (0.0, 17.3)	**22.0** (14.2, 29.8)
		Disagree	**43.2** (27.2, 59.2)	**47.2** (30.9, 63.5)	**58.7** (49.5, 67.9)
		Don’t know	**51.4** (35.3, 67.5)	**44.4** (28.2, 60.6)	**19.3** (11.9, 26.7)
22. Effects of stress on memory are driven primarily by autonomic nervous system activity.*	*primarily ANS activity*	Agree	**37.8** (22.2, 53.4)	**27.8** (13.2, 42.4)	
		Disagree	**13.5** (2.5, 24.5)	**58.3** (42.2, 74.4)	
		Don’t know	**48.6** (32.5, 64.7)	**13.9** (2.6, 25.2)	
23. Encoding is facilitated when the autonomic nervous system is activated while experiencing an emotional event such as a crime.*	*ANS facilitates*	Agree	**48.6** (32.5, 64.7)	**94.4** (86.9, 100.0)	
		Disagree	**13.5** (2.5, 24.5)	**2.8** (0.0, 8.2)	
		Don’t know	**37.8** (22.2, 53.4)	**2.8** (0.0, 8.2)	
24. Rapid nongenomic glucocorticoids have a beneficial effect on memory formation for an event such as a crime.*	*rapid cortisol is beneficial*	Agree	**13.5** (2.5, 24.5)	**52.8** (36.5, 69.1)	
		Disagree	**2.7** (0.0, 7.9)	**11.1** (0.8, 21.4)	
		Don’t know	**83.8** (71.9, 95.7)	**36.1** (20.4, 51.8)	
25. Slow genomic glucocorticoids have a detrimental effect on memory formation for an event such as a crime.*	*slow cortisol is detrimental*	Agree	**13.5** (2.5, 24.5)	**27.8** (13.2, 42.4)	
		Disagree	**2.7** (0.0, 7.9)	**30.6** (15.5, 45.7)	
		Don’t know	**83.8** (71.9, 95.7)	**41.7** (25.6, 57.8)	
26. At encoding, noradrenergic stimulation alone can be sufficient for enhancing the connectivity and excitability within brain networks related to memory.*	*noradrenergic alone*	Agree	**21.6** (8.3, 34.9)	**41.7** (25.6, 57.8)	
		Disagree	**10.8** (0.8, 20.8)	**19.4** (6.5, 32.3)	
		Don’t know	**73.0** (58.7, 87.3)	**38.9** (23.0, 54.8)	
27. At encoding, glucocorticoid actions alone can be sufficient for enhancing the connectivity and excitability within brain networks related to memory.*	*glucocorticoid alone*	Agree	**16.2** (4.3, 28.1)	**30.6** (15.5, 45.7)	
		Disagree	**0.8** (0.8, 20.8)	**36.1** (20.4, 51.8)	
		Don’t know	**73.0** (58.7, 87.3)	**33.3** (17.9, 48.7)	
28. To observe the effects of stress during encoding on memory, both the autonomic nervous system and the HPA axis must be activated at the same time.*	*HPA & ANS activated*	Agree	**16.2** (4.3, 28.1)	**27.8** (13.2, 42.4)	
		Disagree	**8.1** (0.0, 16.9)	**36.1** (20.4, 51.8)	
		Don’t know	**75.7** (61.9, 89.5)	**36.1** (20.4, 51.8)	
29. When noradrenergic arousal interacts with nongenomic glucocorticoids during retrieval, memory is typically impaired.*	*HPA & ANS retrieval*	Agree	**13.5** (2.5, 24.5)	**44.4** (28.2, 60.6)	
		Disagree	**0.0**	**11.1** (0.8, 21.4)	
		Don’t know	**86.5** (75.5, 97.5)	**44.4** (28.2, 60.6)	

*Note.* * = statement presented only to expert sample. Agree = somewhat agree + strongly agree. Disagree = somewhat disagree + strongly disagree. Numbers in parentheses = 95% CIs (lower, upper)

## Method

### Participants

We did not conduct a typical power analysis to determine sample size for two reasons: (i) we did not have specific hypotheses for this exploratory survey and (ii) the pool of experts is, naturally, constrained due to the specific nature of expertise. Thus, we based the number of participants on our expected expert response rate estimating with respect to an initial list of experts in relevant areas. We anticipated that we would obtain responses from around 50 eyewitness experts and 50 fundamental memory experts. In the event that such numbers were not forthcoming, our stopping rule was to continue collection for as long as feasible. We planned to recruit a similar number of laypeople, thus aiming for at least 100 layperson participants between the ages of 18 and 65 to best reflect age range in a group of potential American jurors. The survey was preregistered on the OSF (https://osf.io/b93px?view_only=f83715544c4640c79c3fbfa50d996154). Table 3 presents the demographic information for both the final laypeople and expert samples.

**Table 3:** Overview of survey participants’ demographics

	Age	Gender	Race/ethnicity*	Nationality	Education	Legal system involvement
Laypeople *N* = 109	*M* = 37.52 *SD* = 10.82 Range: 19 to 65	53.2% male 45.0% female 0.9% nonbinary 0.9% prefer not to say	82.6% White 8.3% Hispanic, Latino, or Spanish origin 5.5% Black or African American 6.4% Asian 0.9% American Indian or Alaskan Native	100% American	57.8% Bachelor’s 26.6% High school 11.0% Master’s 3.7% Other (e.g.,Associate’s degree orsome college)	Been on a jury: 15.6% Witnessed a crime: 34.9% (*n* = 21 for violent crime, *n* = 17 for nonviolent crime) Of these, 34.2% (*n* = 13) directlyinvolved in criminal justice system
Experts *N* = 73	*M* = 46.07 *SD* = 15.95 Range: 26 to 87	50.7% male 46.6% female 2.7% prefer not to say	94.5% White 2.7% Hispanic, Latino, or Spanish origin 1.4% Asian 1.4% Middle Eastern or North African 2.7% prefer not to say	64.4% American 11.0% German 9.6% Dutch 5.5% British 2.7% Australian 1.4% Danish 1.4% Canadian 1.4% Spanish 1.4% Italian 1.4% French	89.0% Doctorate 11.0% Master’s Degree in: 91.8% Psychology 5.5% Other science 2.7% Medicine	Acted as expert witness**: 69.9% Never 28.8% More than once

*Note.* * = multiple choices possible. ** = one response missing

#### Exclusion criteria

We included four attention checks. Specifically, within the instructions, we informed participants that at the end of the survey, they would be asked to choose a shape and that they should select *triangle*. In addition, we included three unrelated mock statements with a clear answer (e.g., *Most humans live more than two hundred years*). We excluded participants who failed more than one attention check. Participants were also excluded if they completed the survey in under 3 minutes. On average, laypeople completed the survey in 9.21 min (*SD* = 6.64) and experts in 23.37 min (*SD* = 19.21).^1^

#### Laypeople

We recruited 129 American participants using Amazon Mechanical Turk (MTurk), an online crowdsourcing marketplace. MTurk has been shown to be a viable platform for academic data collection when compared with other commonly used platforms (Kees, Berry, Burton, & Sheehan, 2017). We selected this platform because of the ease and speed of data collection, but also to reach a broad sample of individuals who may indeed be potential jurors (i.e., the general American public). We excluded 20 laypeople because they did not pass three of the four attention checks (*n* = 15), they were older than our cutoff age of 65 (*n* = 4), or they completed the survey too quickly (*n* = 1), leaving us with a layperson sample of *N* = 109. Laypersons were thanked and received $1 as compensation. These data were collected within one week in September 2019.

#### Experts

Following earlier surveys (Kassin et al., 1989; Kassin et al., 2001), we contacted eligible experts that we identified by perusing the pertinent literature to find those who had published peer-reviewed papers on this topic (i.e., eyewitness and fundamental memory research related to stress and memory). To do so, we searched for combinations of related terms (e.g., *stress*, *arousal*, *emotional better remembered*, *memory*, *eyewitness*) on relevant databases (e.g., PsycInfo). Additionally, we examined publications referenced in larger meta-analyses examining stress effects on memory (e.g., Deffenbacher et al., 2004; Shields et al., 2017). Finally, we separately made a list of experts in the field of stress and memory and searched for additional research that was published by them related to emotion/stress/arousal and memory. We sent one initial email and two follow-up emails to 150 researchers over a 4-month period between May and September 2019. Additionally, we contacted members of the Society for Applied Research on Memory and Cognition, the European Association of Psychology and Law, the American Psychology-Law Society, and the Stress-NL Consortium^2^ through server emails, explicitly requesting participation from those who had published peer-reviewed articles on the topic of the effects of stress, arousal, or emotion on memory. The survey was closed in November 2019, after more than 6 months of data collection through these multiple avenues. Out of participants responding to the expert survey, eight did not pass the attention checks, and one participant asked to withdraw their data postsurvey.

These self-reported experts received additional demographic questions about their research (see Table 3). Of the final sample, 89% possessed a doctorate degree, and the other 11% held a master’s degree, with 66% of experts expressing the effects of arousal/stress on memory as a primary area of interest. Additionally, these experts had published in scientific journals, law reviews, books, chapters, magazines, or newsletters (*Mdn =* 27*, IQR* = 68, range = 0 to 557), many of which focused specifically on the effects of stress on memory (*Mdn =* 4, *IQR* = 10, range = 0 to 400).^3^ Nearly 29% of experts had acted as an expert witness, sometimes testifying specifically about the effects of stress on memory (*Mdn* = 5, *IQR* = 20, range = 0 to 500).

If experts classified their primary area of research as eyewitness memory, applied memory in forensic contexts, or other related forensic psychological areas, we assigned them to the eyewitness memory expert group. If experts classified their primary area of research as the neuroscience of memory or another memory or related psychological area, we assigned them to the fundamental memory expert group. Two independent researchers categorized the unclassified research areas, resulting in a high degree of reliability (Koo & Li, 2016), ICC (intraclass correlation coefficient; absolute agreement, two-way mixed-effects model) = .866, 95% CI [.709. .942], *F* (21, 21) = 13.952, *p* < .001. Disagreements (*n* = 1) between coders were resolved through discussion. Of the final sample (*N* = 73), 37 were eyewitness experts and 36 were fundamental memory experts. Experts were thanked upon completion, but received no reimbursement.

### Materials

#### Survey

We created the survey using the online platform Qualtrics (Qualtrics, Provo, UT), with separate versions for laypeople and experts. Both survey versions and data sets are accessible on the OSF, with expert demographic information removed to protect confidentiality (https://osf.io/jpra2/?view_only=a87bc3abda8c4cb699299ecfc9cc94d2). After consenting, participants completed the survey in a self-paced format. They were unable to return to any of the questions once they had continued the survey. For each statement, we created a shorthand term for brevity; Table 2 presents these shorthands alongside each statement.

#### Layperson and expert statements

Both survey versions contained the same 21 statements related to the effects of acute stress on (eyewitness) memory. The first fixed statement was a word-for-word reproduction of the single item used in past expert surveys (Kassin et al., 1989; Kassin et al., 2001) for comparison purposes (i.e., *Very high levels of stress impair the accuracy of eyewitness testimony*). We also generated a list of topics relevant to the stress–memory relationship that may be pertinent for eyewitness-related scenarios and are often discussed in relevant reviews or papers (i.e., Christianson, 1992; Deffenbacher et al., 2004; Shields, 2020; Shields et al., 2017). As such, the 20 other statements were generated with reference to past theories or findings about potential effects or moderators of effects or were otherwise relevant to eyewitness-related settings. Specifically, the random-order statements addressed specific issues related to stressor timing on the stress–memory relationship, potential moderators of the stress–memory relationship (e.g., type of memory test, age, role, detail type, stimulus type, stimulus valence), and other areas of interest to the eyewitness field (e.g., misinformation effects, memory specificity, relation to repressed memories). Some of the statements better agreed with the state of the science (e.g., *stress impairs retrieval*; see Shields et al., 2017), others were less established (e.g., *children less affected*; see Deffenbacher et al., 2004, for a discussion), debated (e.g., *stress enhances encoding*), or overlooked in past research (e.g., *short crime*).

We asked experts and laypeople to rate each statement from a list of options. Similar to past surveys (e.g., Akhtar et al., 2018; Magnussen, Melinder, Stridbeck, & Raja, 2010; Read & Desmarais, 2009), experts and laypeople chose from one of five options: *strongly disagree*, *somewhat disagree*, *somewhat agree*, *strongly agree*, or *don’t know*. Instructions at the beginning of the survey discouraged guessing and clarified to experts that a *don’t know* choice was appropriate when the current research in the field is inconclusive. For the final analysis, answers were collapsed and coded as *disagree* (*strongly disagree* and *somewhat disagree*) and *agree* (*strongly agree* and *somewhat agree*; cf. Benton, Ross, Bradshaw, Thomas, & Bradshaw, 2006; Read & Desmarais, 2009). Table A in the supplementary materials shows the distribution of results across all five response categories.

#### Additional expert statements and questions

The expert version of the survey contained eight additional random-order statements relating to more technical and fundamental topics likely to be unknown and unsuitable for a layperson sample. These statements were generated with reference to current neurobiological theories regarding acute stress effects on memory (e.g., Diamond et al., 2007; Joëls et al., 2006; Quaedflieg & Schwabe, 2018). Specifically, the statements focused on the most relevant physiological stress responses, addressing the precise roles that the autonomic nervous system and glucocorticoid activity play within the stress–memory relationship.

Additionally, for each statement, we asked experts (a) whether they believed the statement was reliable enough for psychologists to present in courtroom testimony (yes or no; *court reliability*); (b) whether their opinion was based on published, peer reviewed, and scientific research (yes or no; *research basis*); and (c) whether they would say that most laypeople believe the statement to be true as a matter of common sense (yes, no, or don’t know; *common sense*). Tables B and C in supplementary materials show responses to these additional questions.

### Data analyses

To address our research questions, we compared (i) expert responses to past expert survey findings, (ii) eyewitness experts to fundamental memory expert responses, and (iii) layperson to expert responses. We conducted a chi-square test between the two groups for each relevant comparison: *endorsements*, referring to whether participants agreed or disagreed with each statement, and *selections*, referring to whether participants agreed/disagreed or selected *don’t know.* We preregistered that we would use a Bonferroni correction and set the alpha to .0017 (.05/29) to correct for multiple comparisons. However, to better preserve power, we instead used a Holm–Bonferroni correction (Holm, 1979) by adjusting *p* values based on the number of tests and comparing with an alpha of .05.

## Results

### Comparison with past work

Following Benton et al. (2006), we compared the proportion of experts who agreed that the statement *Very high levels of stress impair the accuracy of eyewitness testimony* was reliable enough for psychologists to present in court with data from a past expert survey that used the same statement (Kassin et al., 2001). Although 19 years have passed since the 2001 survey, there is a chance that some of the same experts participated in both surveys, which would violate the assumption of independence for a chi-square test. It is not possible to tell whether this is the case, but due to the possibility, we present the results of this preregistered chi-square comparison with caution. The current level of endorsement (61% of experts; i.e., 43 of 71) was similar to the previous survey (60%, i.e., 37 of 62; Kassin et al., 2001), and these endorsement rates did not differ statistically significantly from one another, χ^2^(1, *N* = 133) = 0.011, *p* = .917, φ = .009.

### Eyewitness experts versus fundamental memory experts

**Figure 1:**
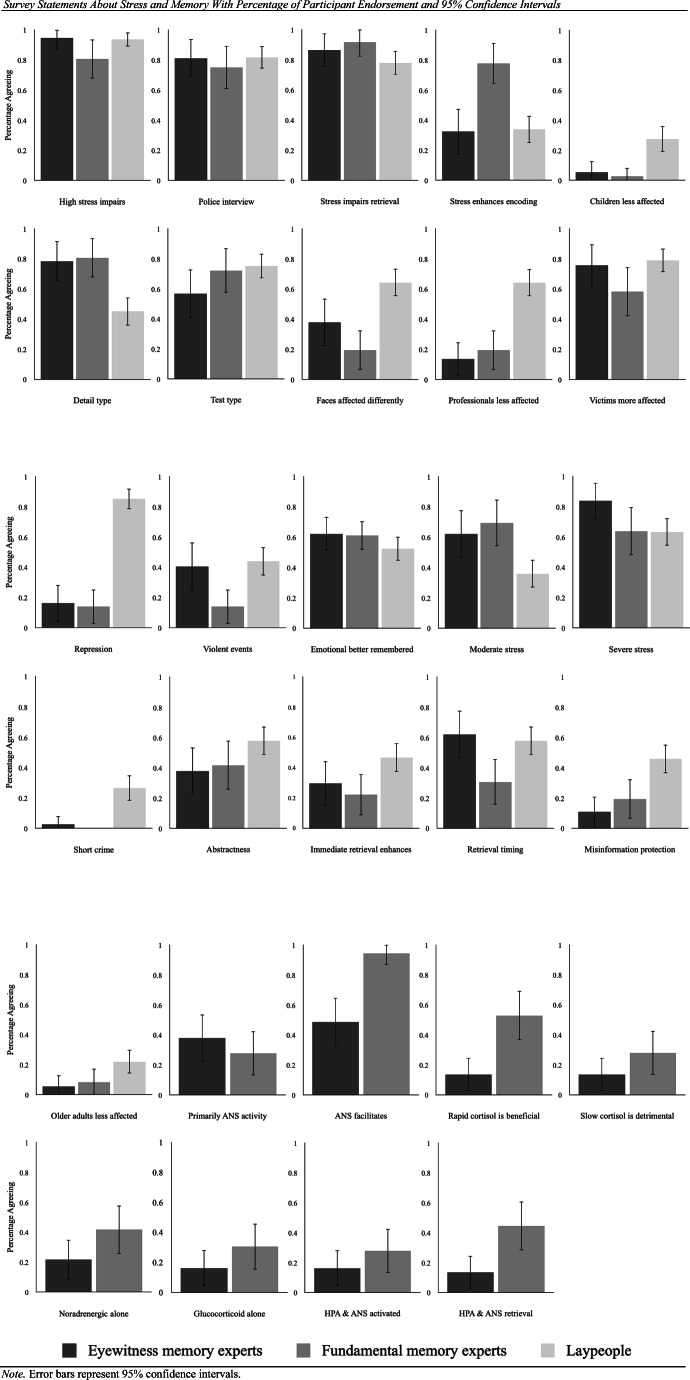
Survey statements about stress and memory with percentage of participant endorsement and 95% confidence intervals

Table 2 presents expert responses and for each statement, categorized by research field. Figure 1 provides a visual overview of agreement rates for each statement between the three groups. We first compared eyewitness memory experts and fundamental memory experts on endorsements (i.e., whether they agreed or disagreed with each statement). Table 4 shows the inferential statistics for these comparisons. A statistically significantly difference between groups *(*φ = .462) emerged for only one statement, *stress enhances encoding*. A greater proportion of fundamental memory experts (77.8%) than eyewitness memory experts (32.4%) agreed with the idea that stress experienced during encoding enhances memory.

**Table 4:** Inferential statistics for 2 × 2 χ^2^ tests comparing endorsements (*agree* versus *disagree*) in eyewitness and fundamental memory experts (*df* = 1)

Statement	*n*	χ^2^	*p*	Adjusted *p*	φ
1. High stress impairs*	71		.055	.084	.241
2. Police interview*	65		.475	.084	.099
3. Stress impairs retrieval*	69		.615	.084	.113
**4. Stress enhances encoding**	**70**	**14.933**	**<.001**	**<.001**	**.462**
5. Children less affected*	50		>.999	.084	.044
6. Detail type*	68		.735	.084	.071
7. Test type*	57		.179	.084	.193
8. Faces affected differently	47	1.978	.160	.084	.205
9. Professionals less affected	70	0.728	.394	.084	.102
10. Victims more affected	63	0.225	.635	.084	.060
11. Repression	64	0.011	.917	.084	.013
12. Violent events	60	3.429	.064	.084	.239
13. Emotional better remembered	60	0.089	.766	.084	.038
14. Moderate stress	61	0.144	.704	.084	.049
15. Severe stress	68	0.949	.330	.084	.118
16. Short crime*	68		>.999	.084	.119
17. Abstractness	61	0.361	.548	.084	.077
18. Immediate retrieval enhances	48	0.426	.514	.084	.094
19. Retrieval timing	56	8.720	.003	.084	.395
20. Misinformation protection*	52		>.999	.084	.022
21. Older adults less affected*	38		>.999	.084	.057
22. Primarily ANS activity	50	8.099	.004	.084	.402
23. ANS facilitates*	58		.032	.084	.303
24. Rapid cortisol is beneficial*	29		>.999	.084	.008
25. Slow cortisol is detrimental*	27		.182	.084	.299
26. Noradrenergic alone*	33		>.999	.084	.047
27. Glucocorticoid alone	34	0.567	.452	.084	.129
28. HPA & ANS activated*	32		.433	.084	.209
29. HPA & ANS retrieval*	25		.549	.084	.218

*Note*. Adjusted *p* = Holm–Bonferroni adjustment for multiple comparisons. **Bold** = adjusted *p* significant at the .05 level. * = Fisher’s exact test instead of chi-square test (when expected cell sizes <5)

For this statement, we also explored relevant data for notable descriptive findings. First, we looked at the broader breakdowns of responses across all five categories (reported in Table A in supplementary materials). The majority of the fundamental memory group selected *somewhat agree* (63.9%), with the rest selecting *strongly agree* (13.9%) or *somewhat disagree* (19.4%), and only one electing *don’t know* (2.8%). On the other hand, eyewitness experts showed a wider distribution: 8.1% selected *strongly agree*, 24.3% *somewhat agree*, 37.8% *somewhat disagree*, 24.3% *strongly disagree*, and 5.4% *don’t know*. Although the main difference between the groups is clear, examining these broader responses shows the variability in the answers from eyewitness experts in particular. Additionally, we descriptively examined proportions for *stress enhances encoding* only for experts who reported that they had previously testified in court (*n* = 21, of which 18 were eyewitness memory experts and three were fundamental memory experts). These experts were mostly eyewitness memory experts (85.7%), and we still see large diversity in responding to *stress enhances encoding* in this subgroup. Specifically, experts who had previously testified in court were split on their responses to *stress enhances encoding*: 14.3% strongly agreed, 23.8% somewhat agreed, 42.9% somewhat disagreed, and 19.0% strongly disagreed.

We next examined group differences between eyewitness memory experts and fundamental memory experts on selections (i.e., whether they selected *don’t know* compared with *agree* or *disagree*; see Table D in the supplementary materials). Eight statements differed statistically significantly between groups: *misinformation protection* (φ = .385), *primarily ANS activity* (φ = .374), *ANS facilitates* (φ = .434), *rapid cortisol is beneficial* (φ = .487), *slow cortisol is detrimental* (φ = .436), *glucocorticoid alone* (φ = .397), *HPA & ANS activated* (φ = .399), and *HPA & ANS retrieval* (φ = .443). For each of these statements, a greater proportion of eyewitness memory experts selected *don’t know* than fundamental memory experts.

### Laypeople versus experts

Table 2 presents layperson responses for each statement. Table 5 shows the inferential statistics for the endorsement comparisons between laypeople and experts. The two groups differed statistically significantly in their responses to eight of the statements. A greater proportion of laypeople agreed with the statements compared with experts for six of these statements: *children less affected (*φ = .301), *faces affected differently* (φ = .416), *professionals less affected* (φ = .506), *repression* (φ = .756), *short crime* (φ = .396), and *misinformation protection* (φ = .355). For *detail type* (φ = .316) and *moderate stress* (φ = .366), a greater proportion of experts agreed with the statements compared with laypeople.

**Table 5:** Inferential Statistics for 2 × 2 χ^2^ tests comparing endorsements (*agree* versus *disagree*) in experts and laypeople (*df* = 1)

Statement	*n*	χ^2^	*p*	Adjusted *p*	φ
1. High stress impairs*	177		>.999	.078	.124
2. Police interview	168	0.058	.810	.078	.019
3. Stress impairs retrieval	168	2.959	.085	.078	.133
4. Stress enhances encoding	165	5.361	.021	.078	.180
**5. Children less affected**	**142**	**12.857**	**<.001**	**<.001**	**.301**
**6. Detail type**	**156**	**15.613**	**<.001**	**<.001**	**.316**
7. Test type	150	0.959	.327	.078	.080
**8. Faces affected differently**	**130**	**22.472**	**<.001**	**<.001**	**.416**
**9. Professionals less affected**	**172**	**44.109**	**<.001**	**<.001**	**.506**
10. Victims more affected	163	1.837	.175	.078	.106
**11. Repression**	**165**	**94.295**	**<.001**	**<.001**	**.756**
12. Violent events	148	6.463	.011	.078	.209
13. Emotional better remembered	149	1.992	.158	.078	.116
**14. Moderate stress**	**155**	**20.789**	**<.001**	**<.001**	**.366**
15. Severe stress	163	0.984	.321	.078	.078
**16. Short crime**	**156**	**24.480**	**<.001**	**<.001**	**.396**
17. Abstractness	151	7.704	.006	.078	.226
18. Immediate retrieval enhances	134	5.612	.018	.078	.205
19. Retrieval timing	145	1.575	.210	.078	.104
**20. Misinformation protection**	**135**	**19.721**	**<.001**	**<.001**	**.382**
21. Older adults less affected	126	2.984	.084	.078	.154

*Note*. Adjusted *p* = Holm–Bonferroni adjustment for multiple comparisons. **Bold** = adjusted *p* significant at the .05 level. * = Fisher’s exact test instead of chi-square test (when expected cell sizes <5)

For these statistically significant statements, we also descriptively examined responses on a broader scale (see Table A in supplementary materials) to explore noteworthy differences. For example, although 20.2% of laypeople selected *strongly agree* for *faces affected differently*, the majority of experts stuck with a middle category (*somewhat agree:* 23.3%, *somewhat disagree:* 28.8%*)* or selected *don’t know* (35.6%), highlighting the lack of strong expert beliefs about this statement. In addition, 0% of experts strongly agreed on *professionals less affected*, while around a quarter of laypeople expressed this extreme agreement (26.6%). Similarly, for *repression*, 4.1% of experts but 33.9% of laypeople selected *strongly agree*, while 57.5% of experts and 1.8% of laypeople selected *strongly disagree*. Examining the extreme ends of the broader response scale highlights the extent of the dissimilarity between groups for these statements.

We also examined differences between layperson and expert selections (see Table E in the supplementary materials). For only one statement did the distribution differ statistically significantly between groups, indicating that a greater proportion of experts selected *don’t know* for the *older adults less affected* statement than laypeople, χ^2^(1, *N* = 182) = 16.88, *p* < .001, φ = .305.

## Discussion

In this survey study, we gathered beliefs from memory experts and laypeople related to the effects of stress on eyewitness memory. We were primarily interested in proportions of each group who agreed, disagreed, or selected *don’t know* for each statement (see Table 2). Additionally, we compared endorsements and selections between groups. In line with previous surveys, we found that most experts in this sample strongly endorsed the belief that high levels of stress impair the accuracy of eyewitness testimony (e.g., Kassin et al., 1989; Kassin et al., 2001; Yarmey & Jones, 1983). In addition, both groups strongly endorsed the statement that stress during retrieval impairs memory, which is in line with findings from fundamental research (e.g., Shields et al., 2017; Wolf, 2017). However, when examining more specific statements in regard to encoding (*stress enhances encoding*) and retrieval (*stress impairs retrieval*), we saw a divergence between eyewitness and fundamental memory experts. Fundamental memory experts generally agreed that experiencing stress at encoding enhances memory, whereas eyewitness memory experts did not.

Prior research examining the effects of stress during encoding on memory is mixed, with different results often emerging across research fields (e.g., S. D. Davis, Peterson, Wissman, & Slater, 2019; Deffenbacher et al., 2004, vs. Henckens et al., 2009; Hoscheidt, LaBar, Ryan, Jacobs, & Nadel, 2014; Vogel & Schwabe, 2016). These contrasting findings perhaps account for the contradictory understandings about the effects of encoding stress on memory that emerged in this survey, and are likely due to methodological differences between the research fields. Fundamental memory research tends to use robust experimental methodology including validated laboratory stressors to induce acute stress (e.g., Trier Social Stress Test; Kirschbaum, Pirke, & Hellhammer, 1993), physiological and subjective manipulation checks to confirm stress inductions, and sufficient retention intervals between sessions (i.e., at least 24 hours) to distinguish the stress effects of encoding and retrieval on memory performance. However, these fundamental studies often examine memory performance for more basic types of stimuli (e.g., word lists, static pictures; Schwabe, Bohringer, Chatterjee, & Schachinger, 2008; Smeets, Giesbrecht, Jelicic, & Merckelbach, 2007; Zoladz et al., 2011).

On the other hand, eyewitness memory laboratory research uses unvalidated stressors such as violent videos, electric shocks, or self-reports (e.g., Bailis & Mueller, 1981; Brigham, Maass, Martinez, & Wittenberger, 1983; Clifford & Hollin, 1981; Kramer, Buckhout, & Eugenio, 1990). Additionally, many eyewitness experiments rely only on self-reported stress as a manipulation check for the stress induction (e.g., Buckhout, Alper, Chern, Silverberg, & Slomovits, 1974; S. D. Davis et al., 2019). Indeed, as pointed out by Sauerland et al. (2016), only seven studies included in the Deffenbacher et al. (2004) meta-analysis report physiological stress measures. Subjective reports of stress, however, do not always correlate with physiological acute stress responses (Hellhammer & Schubert, 2012). Eyewitness field studies show similar limitations, failing to confirm HPA-axis activation (i.e., by examining cortisol) or lacking a sufficient retention interval to specifically examine effects of encoding stress on memory performance (e.g., Hope et al., 2016; Hulse & Memon, 2006; Morgan et al., 2004; Valentine & Mesout, 2008). The single session designs often used in eyewitness memory research make it impossible to isolate the effects of encoding stress on different memory phases (i.e., consolidation versus retrieval; Sauerland et al., 2016; Thomas & Karanian, 2019).

Many of these methodological differences between fields stem from the distinct goals of each particular research field. While the fundamental memory field often aims to examine the basic neurobiological activities underlying the stress–memory relationship, the eyewitness memory field is more interested in the impact that acute stress can have on memory for a crime in applied witness contexts. Thus, the eyewitness memory field notably attempts to mimic witness experiences. However, in such applied experiments, isolating stress effects can be difficult, sometimes leading to a mischaracterization and overgeneralization of the term *acute stress* (i.e., a physiological response involving HPA axis activation, as defined in the fundamental memory field). Stemming from these unique research aims, the varied methodology between fields likely contributes to the contrasting results, and perhaps explains why experts from the two fields often express opposing views about how encoding stress affects memory performance. This divergence in perspectives suggests an absence of interactions between research fields. Critically, understanding results from fundamental memory studies that use more precise methodology might be useful for eyewitness experts. Eyewitness researchers examining the effects of stress on memory performance should strive to gain knowledge about the fundamental stress literature and the methodological gold standards (see Shields, 2020), and should also aim to collaborate with fundamental stress experts. In addition, fundamental memory researchers could conduct research alongside or in consultation with eyewitness memory researchers to produce work that better reflects conditions in the real world—for example, by using more ecologically valid scenarios (e.g., mock crimes).

### Expert beliefs about moderators between encoding stress and memory

To better parse responses to the more general statements about encoding stress effects on memory performance, we also probed for experts’ beliefs about potential moderating factors that may affect the relationship between encoding stress and memory. Many of the statements that showed low levels of expert endorsement (i.e., below 50%; *abstractness*, *faces affected differently, violent events, children less affected*, *older adults less affected*) have not been thoroughly empirically tested. For example, although some findings indicate that children (Deffenbacher et al., 2004) or older adults (Hidalgo, Pulopulos, & Salvador, 2019; Smith, Dijkstra, Gordon, Romero, & Thomas, 2019) may be less affected by stress than younger adults, the vast majority of studies have focused solely on younger adults. Therefore, the lack of consensus and higher levels of *don’t know* responses are in line with available research findings. These data may help guide future research by emphasizing some of the moderators that need to be further examined with empirical work. However, some statements received high levels of endorsement despite ambiguity in research findings. There are conflicting findings regarding differences in stress effects on victims versus bystander eyewitnesses (e.g., Hope et al., 2016; Hosch & Bothwell, 1990; Kassin, 1984), yet most of the eyewitness memory experts and fundamental memory experts endorsed the idea that a victim’s memory will be more affected by encoding stress (*victims more affected*). Similarly, the vast majority of both expert groups disagreed that stress experienced during a short crime will not affect memories (*short crime*), although we have not been able to identify any empirical research conducted on this specific topic. Furthermore, both groups generally agreed that eyewitnesses who experience moderate levels of stress during a crime display better levels of memory than those who experience low levels of stress (*moderate stress*). Most experts from both groups also endorsed the idea that severe but not moderate levels of stress generally harm eyewitness memory (*severe stress*). Neuroscientific research supports this inverted-U-shape idea, which suggests poorer cognitive performance at low and high levels of stress and better performance at medium levels of stress (e.g., Abercrombie, Kalin, Thurow, Rosenkranz, & Davidson, 2003; de Kloet, Oitzl, & Joëls, 1999; Lupien, Maheu, Tu, Ficco, & Schramek, 2007). This inverted U might also explain the different findings between the eyewitness and fundamental memory fields. For example, some fundamental memory research suggests that stress induced in the laboratory during encoding enhances stressor-related memory (e.g., Vogel & Schwabe, 2016), while field studies have found impairments in stressor-related memory (e.g., Metcalfe et al., 2019). Although research directly supporting the statements discussed in this section is not substantial, experts might have drawn from relevant theories to support their choices on these topics (e.g., *dual mode model*, *temporal dynamics model*, *Yerkes-Dodson law*; Diamond et al., 2007; Joëls et al., 2006; Yerkes & Dodson, 1908). Empirical work on these generally endorsed but underresearched topics would also be beneficial for understanding the intricacies of the stress–memory relationship.

Other relevant factors endorsed by experts have a more solid research evidence base. For example, both expert groups agreed that emotional stressful experiences are remembered better than nonemotional ones (*emotional better remembered*), an account supported by research (Cahill, Gorski, & Le, 2003; Kuhlmann, Piel, & Wolf, 2005; Shields et al., 2017; but see Schwabe et al., 2008; Shermohammed, Davidow, Somerville, & Murty, 2019). Additionally, both agreed that encoding stress enhances memory for central details and undermines memory for peripheral details (*detail type*). These opinions are generally supported by research that suggests simultaneous helping and harming effects of stress on different types of details (Christianson, 1992; Christianson & Loftus, 1987; Heuer & Reisberg, 1990; but see Lanciano & Curci, 2011; Wessel, van der Kooy, & Merckelbach, 2000). Eyewitness memory experts likely related this statement to the *weapon focus effect* (e.g., Kramer et al., 1990; Loftus, Loftus, & Messo, 1987), a phenomenon demonstrating that eyewitness memory for faces and other details is poorer if a weapon was present during a crime (Fawcett, Fawcett, Peace, & Christie, 2013). Finally, the majority of both groups disagreed that those who experience stress are more likely to have repressed memories than those who do not (*repression*), which is in fact not supported by empirical data (e.g., Otgaar et al., 2019).

### Expert beliefs about moderators between retrieval stress and memory

We also examined factors relevant to stress effects at memory retrieval. The majority of experts who agreed that retrieval stress impairs memory also endorsed a more applied version of this statement, though to a lesser extent. This more applied statement (*police interview*) stems logically from the broader statement (*stress impairs retrieval*), though specific research has not yet been conducted on this topic. Other retrieval-related statements were based on limited prior research. For example, some research suggests that free recall is impaired more than recognition ability by stress before retrieval (*test type*; de Quervain et al., 2003; de Quervain, Roozendaal, Nitsch, McGaugh, & Hock, 2000; Gagnon & Wagner, 2016), a statement generally endorsed by both groups. Some experimental results also suggest that if memory is tested immediately after a stressor, memory is not harmed, but rather is sometimes even enhanced (*immediate retrieval enhances*; Schönfeld, Ackermann, & Schwabe, 2014; Schwabe & Wolf; 2014). However, less than a third of both expert groups agreed. Finally, around two thirds of eyewitness memory experts and one third of fundamental memory experts believed that memory tested 2 hours after a stressor will be worse than memory tested 30 minutes after a stressor (*retrieval timing*), a statement based on some limited results (e.g., Schwabe & Wolf, 2014). These statements have some basis in research but lack a substantial literature, which may explain the absence of expert consensus in this sample.

### Expert beliefs on neuroscientific statements

Experts answered eight additional statements about neuroscientific explanations of stress effects on memory. These statements were mostly based on theoretical research (e.g., Diamond et al., 2007; Joëls et al., 2011; Joëls et al., 2006; Quaedflieg & Schwabe, 2018; Roozendaal, 2002; Schwabe et al., 2012) that delineate the specific timing and roles the autonomic nervous system and glucocorticoid activity play in the relationship between stress and memory. Overall, eyewitness memory experts selected *don’t know* more often than fundamental memory experts for each statement. This disparity between expert groups suggests that eyewitness memory experts understand less about the neuroscience behind the stress–memory relationship. That being said, perhaps most striking in regard to the eight neuroscientific statements was the proportion of *don’t know* selections across *both* groups of experts. Over a third of fundamental experts also selected *don’t know* for most of the neuroscientific statements. Some statements had a more limited research basis, including statements about how noradrenergic stimulation and glucocorticoid activation act specifically alone or together to affect brain networks related to memory (*noradrenergic alone*, *glucocorticoid alone*, *HPA & ANS activated*). Other statements were more established (see Joëls et al., 2006; Quaedflieg & Schwabe, 2018), but did not receive a majority endorsement from fundamental memory experts (*slow cortisol is detrimental*, *HPA & ANS retrieval*). The proportion of *don’t know* selections indicate a lack of knowledge in this research area, suggesting that certain topics are not yet established and accepted by an expert majority—at least in these two research domains.

A majority of fundamental memory experts generally showed consensus on three statements, which point towards research findings that are more accepted. Fundamental memory experts mostly disagreed that effects of stress on memory are *primarily* driven by autonomic nervous system activity, though nearly all agreed that encoding is facilitated when the autonomic nervous system is activated while experiencing an emotional event such as a crime. Additionally, most fundamental memory experts agreed that rapid nongenomic glucocorticoids have a beneficial effect on memory formation. Considering that 78% of fundamental memory experts agreed that experiencing encoding stress enhances memory, these endorsements of neuroscientific explanations of encoding enhancements are perhaps unsurprising.

### Layperson beliefs about stress effects on memory

As juror opinions about stress effects on memory can also enter the courtroom and may affect decision-making (Bornstein et al., 2008), we examined laypeople’s responses and compared them with experts’ responses. In line with experts, most laypeople agreed that high levels of stress impair eyewitness testimony *(high stress impairs*). In contrast to experts, only about a third of laypeople believed that moderate levels of stress at encoding could enhance memory compared with low levels of stress (*moderate stress*). Thus, laypeople tend to view stress as overwhelmingly negative, with any degree of stress in any memory phase generally impairing memory.

Other differences between laypeople’s and experts’ responses point towards diverging opinions of the public, including the controversial belief that stress causes repressed memories (*repression*, 85%), which research suggests is not the case (e.g., Otgaar et al., 2019). Additionally, the majority of laypeople believed that police officers’ memories are resistant to stress effects, while eyewitness and fundamental memory experts did not (*professionals less affected*), a view more in line with the limited research on this topic (e.g., Stanny & Johnson, 2000). Finally, laypeople also believed that stress affects faces differently than other types of stimuli (*faces affected differently*), contrasting lower endorsement levels from eyewitness memory experts and fundamental memory researchers on this underresearched and inconclusive topic.

Whereas expert beliefs are generally formed from research on these topics in academic settings, laypersons’ beliefs likely stem from intuitive feelings or perceptions about each statement. Given that stress is generally viewed as a negative experience (e.g., Adams, 2016; Becker, 2013), it is unsurprising that laypeople seem to view any degree of stress as harmful, in contrast with expert opinion. Laypeople’s agreement that police officers’ memories can withstand stress is also an evident erroneous, but understandable commonsense belief (e.g., Hope, 2016; Stanny & Johnson, 2000). A related statement used in past surveys showed that low percentages of laypeople (28% and 39%) endorsed the idea that *Police officers and other trained observers are no more accurate as eyewitnesses than is the average person* (e.g., *N* = 111, Benton et al., 2006; *N* = 79, Kassin & Barndollar, 1992, respectively). Taken together, these responses suggest that many laypeople believe that professionals are generally better eyewitnesses who are less influenced by external factors such as stress. Two recent surveys also show that large proportions of participants (59% and 67%, respectively) endorsed the idea that traumatic experiences can be unconsciously repressed for many years and then recovered (*N* = 230 and *N* = 79; Otgaar et al., 2019), a statement similar to *repression* in our survey. Factors such as television and media may influence such beliefs. For example, 75% of students (*N* = 613) who reported hearing about someone recovering a repressed memory said they heard about such a circumstance though television (Golding, Sanchez, & Sego, 1996). Additionally, amount of media exposure to information about repressed memories was positively correlated with beliefs in repressed memories. Thus, perhaps the endorsement of the idea that stress causes repressed memories from laypeople in this sample originally stemmed from media or television exposure. To sum up, as demonstrated in this survey and previous surveys, commonsense beliefs do not always align with expert assessments concerning what the contemporary science suggests (e.g., Benton et al., 2006; Simons & Chabris, 2011).

### Implications for applied legal settings

These data serve as an initial empirical attempt of expert and layperson beliefs about the effects of acute stress on memory performance. Although agreement between expert groups was observed on several statements, the most striking difference between groups pertained to the statement that *stress enhances encoding* (φ = .462), where fundamental memory experts mostly agreed and eyewitness memory experts mostly disagreed. From an exploratory analysis, we also saw a descriptive split among experts who had testified in court. That is, not all testifying experts fell on one side of the belief (i.e., agreeing vs. disagreeing with the statement that *stress enhances encoding*). These results further support the idea that different expert witnesses bring different views into the courtroom. In this way, jurors and judges could hear contrasting statements from opposing expert witnesses, or hear from only one expert witness, who could fall on either side of the belief. If an expert witness strongly endorses the idea that encoding stress enhances memory, jurors could assume that testimony provided by an eyewitness who experienced stress is highly reliable. If an expert witness reports the opposite, jurors may unreasonably disregard the testimony of an eyewitness who experienced stress. Thus, for seemingly irresolute statements such as *stress enhances encoding*, exercising caution in the courtroom is important.

On the other hand, these data also suggest that jurors already likely bring their own conceptions about the effects of stress on memory performance into the courtroom. Understanding these preexisting commonsense beliefs is crucial for knowing where expert witness knowledge is needed. For example, if laypeople assume that any amount of stress will automatically impair memory, they may view the testimony from a stressed eyewitness as lacking in probative value. This could later affect their legal decisions. Similarly, if laypeople are unaware of how stress can affect the memories of professionals such as police officers, they may give too much credence to their testimonies over others. For topics like these that show greater consensus from experts in general, reports from an expert witness could be particularly valuable in the courtroom.

### Limitations and future directions

There are a number of limitations associated with the current survey. Some nuance is lost when using closed statements with an agree/disagree response format that force respondents to ‘choose a side’. Particularly, responding to broad statements (e.g., *high stress impairs, stress enhances encoding*, *stress impairs retrieval*) that use wide-ranging terms such as “memory” can be challenging. That is, “memory” could be thought of quite generally—for example, knowing that an experience occurred (e.g., ability to remember gist or central information) or much more specifically (e.g., ability to remember detailed or peripheral information). For this reason, we included several other statements to provide us with more insight into potential moderators that could explain differences in how the broader statements were answered (e.g., *severe stress*, *detail type*, *abstractness*). With these more specific statements, we believe that, in contrast to previous surveys, we were better able to interpret the results than if we used broad generalizations alone. Future research could explore the use of a less rigid method such as a qualitative survey or perhaps focus groups, to examine the specific circumstances in which experts believe stress enhances, impairs, or does not affect memory.

A potential limitation pertaining to the layperson group is that the statements might have been too technical for them. We initially addressed this by leaving out the technical neuroscientific statements for the lay sample (i.e., items 22–29), and by explicitly defining certain memory jargon, such as terms like *encoding* and *retrieval*. However, other terms may have also been too technical for them to fully understand the statements, such as understanding the meaning behind central versus peripheral details or free recall versus recognition. Given the laypeople’s responses for these statements, it does not seem that much concern is warranted, although future research should aid layperson understanding as much as possible in surveys.

Our expert sample size is comparable to past expert surveys (e.g., Kassin et al., 1989; Kassin et al., 2001). Nonetheless, analyses comparing expert groups might be underpowered due to the limited number of experts who were involved in the survey. Obtaining this expert sample was difficult due to the inherently limited population, and we collected expert data for six months using multiple channels and repeated calls in an attempt to access the largest sample possible. We also attempted to reduce the Type I error by using a Holm–Bonferroni correction. With a final sample of 73 experts, the between-expert comparisons are only powered to detect large effects (80% powered to detect Cohen’s *w* = .50), whereas the expert–layperson analyses (*n* = 182) are powered to detect medium and large samples (80% powered to detect Cohen’s *w* = .30). Thus, results should be interpreted with caution, keeping the limited sample size and number of comparisons in mind. Specifically, owing to the reduced power, nonstatistically significant differences may have been due to an inability detect smaller effect sizes, and the statistically significant differences may be overestimated. On the other hand, large effects may be most relevant for real-world application in this area.

In addition to a greater number of experts, a more comprehensive representation of possible experts would also benefit future research. Our study examined only a subsection of potential experts: academics who investigate stress effects on memory or related topics. Nearly a third of experts in this survey have experience acting as expert witnesses in court. However, other categories of people who also testify in court settings as experts (e.g., clinical psychologists) may not publish on these matters. This survey did not include those various groups, and thus our definition of expert is restrained to those working and publishing in academic contexts.

The results from this survey might serve as a beneficial guide for future research in this area. Statements that experts answered with *don’t know* or where *agree* and *disagree* selections were divided may indicate areas that need to be better investigated. These areas include factors such as age differences (*children less affected*, *older adults less affected*), specificity of stressor timing (*immediate retrieval enhances*, *retrieval timing*), factors at encoding (*violent events*, *moderate stress*), and the form of remembered information (*test type*, *faces affected differently*). The results also show the continuing need for fundamental neuroscientific research about how biological stress responses affect memory formation and retrieval in humans. Finally, we suggest that academic experts should be aware of research that exists across the wider research domain, particularly if they plan to testify in court on these matters.

## Conclusion

This survey explored contemporary experts’ and laypeople’s beliefs about the effects of stress on memory encoding and retrieval. Only five statements (i.e., *high stress impairs*, *stress impairs retrieval*, *police interview*, *detail type*, and *short crime*) out of 29 received consensus levels of over 75% among both eyewitness and fundamental memory expert groups. As such, these results appear to indicate a general lack of consensus about most factors that play a role in the stress–memory relationship. However, the two expert groups only statistically differed from each other regarding the enhancing effects of encoding stress on memory. Examining beliefs about other factors, such as stress severity and type of remembered detail, provided some insight into this disparity. Laypeople differed from experts on some factors and endorsed some ideas that are not supported by empirical research—for example, that trained professionals such as police are less affected by stress and that stress causes repressed memories. In summary, results from this survey suggest that whereas some factors have a wide consensus among experts, there may be significant gaps in this literature where more research is needed to enhance our understanding of the relationship between stress and memory.

## Supplementary Information

ESM 1 (DOCX 55 kb)

## Footnotes

1 Result reported here excludes three outliers in the expert group (152 hours, 16 hours, and 10 hours) who likely left the survey tab open on their computer during completion over 1 or several days.

2 We did not originally include the Stress-NL Consortium in our preregistered plan for survey dissemination, but decided to distribute the survey to this organization due to its contact with current stress experts.

3 Analyses were also conducted without the participant reporting zero publications (*n* = 1) and those with missing numerical responses (*n* = 2), and results remained the same.

## References

[CR1] Abercrombie HC, Kalin NH, Thurow ME, Rosenkranz MA, Davidson RJ (2003). Cortisol variation in humans affects memory for emotionally laden and neutral information. Behavioral Neuroscience.

[CR2] Adams, T. (2016, February 14). Is there too much stress on stress? *The Guardian*. Retrieved from https://www.theguardian.com/society/2016/feb/14/workplace-stress-hans-selye

[CR3] Akhtar S, Kalin NH, Thurow ME, Rosenkranz MA, Davidson MA (2018). The ‘common sense’ memory belief system and its implications. The International Journal of Evidence & Proof.

[CR4] Bailis KL, Mueller JH (1981). Anxiety, feedback, and self-reference in face recognition. Motivation and Emotion.

[CR5] Becker D (2013). *One nation under stress: The trouble with stress as an idea*.

[CR6] Benton TR, Ross DF, Bradshaw E, Thomas WN, Bradshaw GS (2006). Eyewitness memory is still not common sense: Comparing jurors, judges and law enforcement to eyewitness experts. Applied Cognitive Psychology.

[CR7] Bornstein BH, Hullman G, Miller MK, Miller MK, Bornstein BH (2013). Stress, trauma, and wellbeing in the legal system: Where do we go from here?. *Stress, trauma, and wellbeing in the legal system*.

[CR8] Bornstein BH, O’Bryant S, Zickafoose D (2008). Intuitions about arousal and eyewitness memory: Effects on mock jurors’ judgments. Law & Psychology Review.

[CR9] Bornstein BH, Robicheaux TR (2009). Methodological issues in the study of eyewitness memory and arousal. Creighton Law Review.

[CR10] Brigham JC, Maass A, Martinez D, Wittenberger G (1983). The effect of arousal on facial recognition. Basic and Applied Social Psychology.

[CR11] Buckhout R, Alper A, Chern S, Silverberg G, Slomovits M (1974). Determinants of eyewitness performance on a lineup. Bulletin of the Psychonomic Society.

[CR12] Cahill L, Gorski L, Le K (2003). Enhanced human memory consolidation with post-learning stress: Interaction with the degree of arousal at encoding. Learning & Memory.

[CR13] Christianson S-A (1992). Emotional stress and eyewitness memory: A critical review. Psychological Bulletin.

[CR14] Christianson S-A, Loftus EF (1987). Memory for traumatic events. Applied Cognitive Psychology.

[CR15] Clifford BR, Hollin CR (1981). Effects of the type of incident and the number of perpetrators on eyewitness memory. Journal of Applied Psychology.

[CR16] Conway MA, Justice LV, Morrison CM (2014). Beliefs about autobiographical memory. The Psychologist.

[CR17] Davis, J. A. (2016, June 22). Critical incident stress reactions from violent crime. *Psychology Today.* Retrieved from https://www.psychologytoday.com/us/blog/crimes-and-misdemeanors/201606/critical-incident-stress-reactions-violent-crime

[CR18] Davis SD, Peterson DJ, Wissman KT, Slater WA (2019). Physiological stress and face recognition: Differential effects of stress on accuracy and the confidence–accuracy relationship. Journal of Applied Research in Memory and Cognition.

[CR19] de Kloet ER, Oitzl MS, Joëls M (1999). Stress and cognition: Are corticosteroids good or bad guys?. Trends in Neurosciences.

[CR20] de Quervain DJ-F, Henke K, Aerni A, Treyer V, McGaugh JL, Berthold T, Hock C (2003). Glucocorticoid-induced impairment of declarative memory retrieval is associated with reduced block flow in the medial temporal lobe. European Journal of Neuroscience.

[CR21] de Quervain DJ-F, Roozendaal B, Nitsch RM, McGaugh JL, Hock C (2000). Acute cortisone administration impairs retrieval of long-term declarative memory in humans. Nature Neuroscience.

[CR22] Deffenbacher KA, Bornstein BH, Penrod SD, McGorty EK (2004). A meta-analytic review of the effects of high stress on eyewitness memory. Law and Human Behavior.

[CR23] Deffenbacher KA, Loftus EF (1982). Do jurors share a common understanding concerning eyewitness behavior?. Law and Human Behavior.

[CR24] Dellapaolera, K. S. (2019). *How does stress at time of identification affect eyewitness memory?* (Doctoral dissertation). Retrieved from ProQuest Dissertations and Theses Global database. (ProQuest No. 22582828)

[CR25] Diamond DM, Campbell AM, Park CR, Halonen J, Zoladz PR (2007). The temporal dynamics model of emotional processing: A synthesis on the neurobiological basis of stress-induced amnesia, flashbulb and traumatic memories, and the Yerkes-Dodson Law. Neural Plasticity.

[CR26] Fawcett JM, Fawcett E, Peace KA, Christie J (2013). Of guns and geese: A meta-analytic review of the ‘weapon focus’ literature. Psychology, Crime, & Law.

[CR27] Gagnon SA, Wagner AD (2016). Acute stress and episodic memory: Neurobiological mechanisms and behavioral consequences. Annals of the New York Academy of Sciences.

[CR28] Golding JM, Sanchez RP, Sego SA (1996). Do you believe in repressed memories?. Professional Psychology: Research and Practice.

[CR29] Hellhammer J, Schubert M (2012). The physiological response to Trier Social Stress Test relates to subjective measures of stress during but not before or after the test. Psychoneuroendocrinology.

[CR30] Henckens MJAG, Hermans EJ, Pu Z, Joëls M, Fernández G (2009). Stressed memories: How acute stress affects memory formation in humans. The Journal of Neuroscience.

[CR31] Heuer F, Reisberg D (1990). Vivid memories of emotional events: The accuracy of remembered minutiae. Memory & Cognition.

[CR32] Hidalgo V, Pulopulos MM, Salvador A (2019). Acute psychosocial stress effects on memory performance: Relevance of age and sex. Neurobiology of Learning and Memory.

[CR33] Holm S (1979). A simple sequentially rejective multiple test procedure. Scandinavian Journal of Statistics.

[CR34] Hope L (2016). Evaluating the effects of stress and fatigue on police officer response and recall: A challenge for research, training, practice and policy. Journal of Applied Research in Memory and Cognition.

[CR35] Hope L, Blocksidge D, Gabbert F, Sauer JD, Lewinski W, Mirashi A, Atuk E (2016). Memory and the operational witness: Police officer recall of firearms encounters as a function of active response role. Law & Human Behavior.

[CR36] Hosch HM, Bothwell RK (1990). Arousal, description and identification accuracy of victims and bystanders. Journal of Social Behavior and Personality.

[CR37] Hoscheidt SM, LaBar KS, Ryan L, Jacobs WJ, Nadel L (2014). Encoding negative events under stress: High subjective arousal is related to accurate emotional memory despite misinformation exposure. Neurobiology of Learning and Memory.

[CR38] Hulse LM, Memon A (2006). Fatal impact? The effects of emotional arousal and weapon presence on police officers’ memories for a simulated crime. Legal and Criminological Psychology.

[CR39] Joëls M, Baram TZ (2009). The neuro-symphony of stress. Nature Reviews Neuroscience.

[CR40] Joëls M, Fernández G, Roozendaal B (2011). Stress and emotional memory: A matter of timing. Trends in Cognitive Sciences.

[CR41] Joëls M, Pu Z, Wiegert O, Oitzl MS, Krugers HJ (2006). Learning under stress: How does it work?. Trends in Cognitive Sciences.

[CR42] Kassin SM (1984). Eyewitness identification: Victims versus bystanders. Journal of Applied Social Psychology.

[CR43] Kassin SM, Barndollar KA (1992). *The psychology of eyewitness testimony: A comparison of experts and prospective jurors*. Journal of Applied Social Psychology.

[CR44] Kassin SM, Ellsworth PC, Smith VL (1989). The ‘general acceptance’ of psychological research on eyewitness testimony: A survey of the experts. American Psychologist.

[CR45] Kassin SM, Tubb VA, Hosch HM, Memon A (2001). On the ‘general acceptance’ of eyewitness testimony research: A new survey of the experts. American Psychologist.

[CR46] Kees J, Berry C, Burton S, Sheehan K (2017). An analysis of data quality: Professional panels, student subject pools, and Amazon’s Mechanical Turk. Journal of Advertising.

[CR47] Kirschbaum C, Pirke K-M, Hellhammer DH (1993). The ‘Trier Social Stress Test’: A tool for investigating psychobiological stress responses in a laboratory setting. Neuropsychobiology.

[CR48] Koo TK, Li MY (2016). A guideline of selecting and reporting intraclass correlation coefficients for reliability research. Journal of Chiropractic Medicine.

[CR49] Kramer TH, Buckhout R, Eugenio P (1990). Weapon focus, arousal, and eyewitness memory: Attention must be paid. Law and Human Behavior.

[CR50] Kuhlmann S, Piel M, Wolf OT (2005). Impaired memory retrieval after psychosocial stress in healthy young men. The Journal of Neuroscience.

[CR51] Lanciano T, Curci A (2011). Memories for emotional events: The accuracy of central and peripheral details. Europe’s Journal of Psychology.

[CR52] Loftus, E. F. (1979). *Eyewitness testimony*. Cambridge, MA: Harvard University Press.

[CR53] Loftus EF, Loftus GR, Messo J (1987). Some facts about “weapon focus”. Law and Human Behavior.

[CR54] Lupien SJ, Maheu F, Tu M, Ficco A, Schramek TE (2007). The effects of stress and stress hormones on human cognition: Implications for the field of brain and cognition. Brain and Cognition.

[CR55] Magnussen S, Melinder A, Stridbeck U, Raja A (2010). Beliefs about factors affecting the reliability of eyewitness testimony: A comparison of judges, jurors and the general public. Applied Cognitive Psychology.

[CR56] Metcalfe J, Brezler JC, McNamara J, Malette G, Vuorre M (2019). Memory, stress, and the hippocampal hypothesis: Firefighters’ recollections of the fireground. Hippocampus.

[CR57] Morgan CA, Hazlett G, Doran A, Garrett S, Hoyt G, Thomas P, Southwick SM (2004). Accuracy of eyewitness memory for persons encountered during exposure to highly intense stress. International Journal of Law and Psychiatry.

[CR58] Noon E, Hollin CR (1987). Lay knowledge of eyewitness behaviour: A British survey. Applied Cognitive Psychology.

[CR59] Otgaar H, Howe ML, Patihis L, Merckelbach H, Lynn SJ, Lilienfeld SO, Loftus EF (2019). The return of the repressed: The persistent and problematic claims of long-forgotten trauma. Perspectives on Psychological Science.

[CR60] Quaedflieg CWEM, Schwabe L (2018). Memory dynamics under stress. Memory.

[CR61] Read JD, Desmarais SL (2009). Lay knowledge of eyewitness issues: A Canadian evaluation. Applied Cognitive Psychology.

[CR62] Robbins TW (1984). Cortical noradrenaline, attention and arousal. Psychological Medicine.

[CR63] Robicheaux, T. R. (2016). *Stress and eyewitness memory: Timing of stressor and association with cortisol stress responding* (Doctoral Dissertation). Retrieved from Dissertation Abstracts International. (Accession No. 2016-26518-083)

[CR64] Roozendaal B (2002). Stress and memory: Opposing effects of glucocorticoids on memory consolidation and memory retrieval. Neurobiology of Learning and Memory.

[CR65] Sauerland M, Raymaekers LHC, Otgaar H, Memon A, Waltjen TT, Nivo M, Smeets T (2016). Stress, stress-induced cortisol responses, and eyewitness identification performance. Behavioral Sciences and the Law.

[CR66] Schmechel RS, O’Toole TP, Easterly C, Loftus EF (2006). Beyond the ken? Testing jurors’ understanding of eyewitness reliability evidence. Jurimetrics.

[CR67] Schönfeld P, Ackermann K, Schwabe L (2014). Remembering under stress: Different roles of autonomic arousal and glucocorticoids in memory retrieval. Psychoneuroendocrinology.

[CR68] Schwabe L, Bohringer A, Chatterjee M, Schachinger H (2008). Effects of prelearning stress on memory for neutral, positive, and negative words: Differential roles of cortisol and autonomic arousal. Neurobiology of Learning and Memory.

[CR69] Schwabe L, Joëls M, Roozendaal B, Wolf OT, Oitzl MS (2012). Stress effects on memory: An update and integration. Neuroscience & Biobehavioral Reviews.

[CR70] Schwabe L, Wolf OT (2014). Timing matters: Temporal dynamics of stress effects on memory retrieval. Cognitive, Affective, & Behavioral Neuroscience.

[CR71] Shermohammed M, Davidow JY, Somerville LH, Murty VP (2019). Stress impacts the fidelity but not strength of emotional memories. Brain and Cognition.

[CR72] Shields GS (2020). Stress and cognition: A user’s guide to designing and interpreting studies. Psychoneuroendocrinology.

[CR73] Shields GS, Sazma MA, McCullough AM, Yonelinas AP (2017). The effects of acute stress on episodic memory: A meta-analysis and integrative review. Psychological Bulletin.

[CR74] Simons DJ, Chabris CF (2011). What people believe about how memory works: A representative survey of the U.S. population. PLOS ONE.

[CR75] Smeets T, Giesbrecht T, Jelicic M, Merckelbach H (2007). Context-dependent enhancement of declarative memory performance following acute psychosocial stress. Biological Psychology.

[CR76] Smith AM, Dijkstra K, Gordon LT, Romero M, Thomas AK (2019). An investigation into the impact of acute stress on encoding in older adults. Aging, Neuropsychology, and Cognition.

[CR77] Stanny CJ, Johnson TC (2000). Effects of stress induced by a simulated shooting on recall by police and citizen witnesses. The American Journal of Psychology.

[CR78] Thomas AK, Karanian JM (2019). Acute stress, memory, and the brain. Brain and Cognition.

[CR79] Ulrich-Lai YM, Herman JP (2009). Neural regulation of endocrine and autonomic stress responses. Nature Reviews Neuroscience.

[CR80] Valentine T, Mesout J (2008). Eyewitness identification under stress in the London Dungeon. Applied Cognitive Psychology.

[CR81] Vogel S, Schwabe L (2016). Stress in the zoo: Tracking the impact of stress on memory formation over time. Psychoneuroendocrinology.

[CR82] Wessel I, van der Kooy P, Merckelbach H (2000). Differential recall of central and peripheral details of emotional slides is not a stable phenomenon. Memory.

[CR83] Wolf OT (2012). Immediate recall influences the effects of preencoding stress on emotional episodic long-term memory consolidation in healthy young men. Stress.

[CR84] Wolf OT (2017). Stress and memory retrieval: Mechanisms and consequences. Current Opinions in Behavioral Sciences.

[CR85] Yarmey AD, Jones HPT, Lloyd-Bostock S, Clifford BR (1983). Is the psychology of eyewitness identification a matter of common sense?. *Evaluating witness evidence*.

[CR86] Yerkes, R. M., & Dodson, J. D. (1908). The relation of strength of stimulus to rapidity of habit formation. *Journal of Comparative Neurology and Psychology, 18*(5), 459–482. 10.1002/cne.920180503

[CR87] Yuille JC, Cutshall JL (1986). A case study of eyewitness memory of a crime. Journal of Applied Psychology.

[CR88] Zoladz PR, Clark B, Warnecke A, Smith L, Tabar J, Talbot JN (2011). Prelearning stress differentially affects long-term memory for emotional words, depending on temporal proximity to the learning experience. Physiology & Behavior.

