# Supplementary material for: The effects of stress on eyewitness memory: A survey of memory experts and laypeople
Source: Mem Cognit. 2020 Nov 25;49(3):401–21. doi: 10.3758/s13421-020-01115-4 (PMC8024237; doi:10.3758/s13421-020-01115-4)
Supplement: Supplementary file 1 — (DOCX 55 kb) [file 13421_2020_1115_MOESM1_ESM.docx]

| **Table A**  *Survey Statements With Full Distribution of Participant Endorsement* | | | | | | | |  |
| --- | --- | --- | --- | --- | --- | --- | --- | --- |
| **Shorthand** | **Percentage of participant endorsement** | | | | |  |  |  |
|  |  | **Eyewitness memory experts**  *n* = 37 | **Fundamental memory experts**  *n* = 36 | **Laypeople**  *n* = 109 | |  |  |  |
| 1. *high stress impairs* | Strongly agree  Somewhat agree  Somewhat disagree  Strongly disagree  Don’t know | 48.6  45.9  2.7  0.0  2.7 | 31.6  44.4  13.9  2.8  2.8 | 65.1  28.4  2.8  0.9  2.8 | |  |  |  |
| 1. *police interview* | Strongly agree  Somewhat agree  Somewhat disagree  Strongly disagree  Don’t know | 45.9  35.1  5.4  2.7  10.8 | 25.0  50.0  8.3  5.6  11.1 | 38.5  43.1  11.9  0.9  5.5 | |  |  |  |
| 1. *stress impairs retrieval* | Strongly agree  Somewhat agree  Somewhat disagree  Strongly disagree  Don’t know | 40.5  45.9  0.0  2.7  10.8 | 47.2  44.4  5.6  2.8  0.0 | 34.9  43.1  11.0  1.8  9.2 | |  |  |  |
| 1. *stress enhances encoding* | Strongly agree  Somewhat agree  Somewhat disagree  Strongly disagree  Don’t know | 8.1  24.3  37.8  24.3  5.4 | 13.9  63.9  19.4  0.0  2.8 | 10.1  23.9  35.8  17.4  12.8 | |  |  |  |
| 1. *children less affected* | Strongly agree  Somewhat agree  Somewhat disagree  Strongly disagree  Don’t know | 2.7  2.7  35.1  37.8  21.6 | 0.0  2.8  30.6  25.0  41.7 | | 13.8  13.8  35.8  21.1  15.6 |  |  |  |
| 1. *detail type* | Strongly agree  Somewhat agree  Somewhat disagree  Strongly disagree  Don’t know | 24.3  54.1  8.1  2.7  10.8 | 27.8  52.8  8.3  8.3  2.8 | | 14.7  30.3  29.4  6.4  19.3 |  |  |  |
| 1. *test type* | Strongly agree  Somewhat agree  Somewhat disagree  Strongly disagree  Don’t know | 16.2  40.5  16.2  2.7  24.3 | 22.2  50.0  8.3  0.0  19.4 | | 34.9  40.4  9.2  0.9  14.7 |  |  |  |
| 1. *faces affected differently* | Strongly agree  Somewhat agree  Somewhat disagree  Strongly disagree  Don’t know | 10.8  27.0  24.3  8.1  29.7 | 0.0  19.4  33.3  5.6  41.7 | | 20.2  44.0  8.3  2.7  23.9 |  |  |  |
| 1. *professionals less affected* | Strongly agree  Somewhat agree  Somewhat disagree  Strongly disagree  Don’t know | 0.0  13.5  51.4  35.1  0.0 | 0.0  19.4  38.9  33.3  8.3 | | 26.6  37.6  21.1  8.3  6.4 |  |  |  |
| 1. *victims more affected* | Strongly agree  Somewhat agree  Somewhat disagree  Strongly disagree  Don’t know | 13.5  62.2  13.5  5.4  5.4 | 11.1  47.2  16.7  2.8  22.2 | | 35.3  43.1  10.1  2.8  8.3 |  |  |  |
| 1. *repression* | Strongly agree  Somewhat agree  Somewhat disagree  Strongly disagree  Don’t know | 5.4  10.8  10.8  64.9  8.1 | 2.8  11.1  19.4  50.0  16.7 | | 33.9  51.4  5.5  1.8  7.3 |  |  |  |
| 1. *violent events* | Strongly agree  Somewhat agree  Somewhat disagree  Strongly disagree  Don’t know | 10.8  29.7  43.2  10.8  5.4 | 0.0  13.9  41.7  13.9  30.6 | | 18.3  25.7  27.5  9.2  19.3 |  |  |  |
| 1. *emotional better remembered* | Strongly agree  Somewhat agree  Somewhat disagree  Strongly disagree  Don’t know | 27.0  35.1  16.2  2.7  18.9 | 22.2  38.9  22.2  0.0  16.7 | | 23.9  28.4  28.4  0.9  18.3 |  |  |  |
| 1. *moderate stress* | Strongly agree  Somewhat agree  Somewhat disagree  Strongly disagree  Don’t know | 13.5  48.6  13.5  5.4  18.9 | 11.1  58.3  8.3  8.3  13.9 | | 9.2  26.6  34.9  15.6  13.8 |  |  |  |
| 1. *severe stress* | Strongly agree  Somewhat agree  Somewhat disagree  Strongly disagree  Don’t know | 27.0  56.8  5.4  10.8  0.0 | 19.4  44.4  13.9  8.3  13.9 | | 27.5  35.8  20.2  3.7  12.8 |  |  |  |
| 1. *short crime* | Strongly agree  Somewhat agree  Somewhat disagree  Strongly disagree  Don’t know | 0.0  2.7  24.3  67.6  5.4 | 0.0  0.0  30.6  61.1  8.3 | | 9.2  17.4  41.3  12.8  19.3 |  |  |  |
| 1. *abstractness* | Strongly agree  Somewhat agree  Somewhat disagree  Strongly disagree  Don’t know | 10.8  27.0  29.7  5.4  27.0 | 8.3  33.3  38.9  139.  5.6 | | 22.0  35.8  20.2  4.6  17.4 |  |  |  |
| 1. *immediate retrieval enhances* | Strongly agree  Somewhat agree  Somewhat disagree  Strongly disagree  Don’t know | 0.0  29.7  27.0  10.8  32.4 | 11.1  11.1  22.2  19.4  36.1 | | 13.8  33.0  25.7  5.5  22.0 |  |  |  |
| 1. *retrieval timing* | Strongly agree  Somewhat agree  Somewhat disagree  Strongly disagree  Don’t know | 27.0  35.1  13.5  2.7  21.6 | 5.6  25.0  36.1  8.3  25.0 | | 19.3  38.5  19.3  4.6  18.3 |  |  |  |
| 1. *misinformation protection* | Strongly agree  Somewhat agree  Somewhat disagree  Strongly disagree  Don’t know | 2.7  8.1  18.9  24.3  45.9 | 0.0  19.4  44.4  25.0  11.1 | | 11.9  33.9  23.9  6.4  23.9 |  |  |  |
| 1. *older adults less affected* | Strongly agree  Somewhat agree  Somewhat disagree  Strongly disagree  Don’t know | 0.0  5.4  27.0  16.2  51.4 | 2.8  5.6  33.3  13.9  44.4 | | 7.3  14.7  31.2  27.5  19.3 |  |  |  |
| 1. *primarily ANS activity** | Strongly agree  Somewhat agree  Somewhat disagree  Strongly disagree  Don’t know | 2.7  35.1  10.8  2.7  48.6 | 2.8  25.0  30.6  27.8  13.9 | |  |  |  |  |
| 1. *ANS facilitates** | Strongly agree  Somewhat agree  Somewhat disagree  Strongly disagree  Don’t know | 10.8  37.8  8.1  5.4  37.8 | 25.0  69.4  2.8  0.0  2.8 | |  |  |  |  |
| 1. *rapid cortisol is beneficial** | Strongly agree  Somewhat agree  Somewhat disagree  Strongly disagree  Don’t know | 0.0  13.5  2.7  0.0  83.8 | 11.1  41.7  8.3  2.8  36.1 | |  |  |  |  |
| 1. *slow cortisol is detrimental** | Strongly agree  Somewhat agree  Somewhat disagree  Strongly disagree  Don’t know | 2.7  10.8  2.7  0.0  83.8 | 5.6  22.2  16.7  13.9  41.7 | |  |  |  |  |
| 1. *noradrenergic alone** | Strongly agree  Somewhat agree  Somewhat disagree  Strongly disagree  Don’t know | 0.0  21.6  5.4  2.4  73.0 | 19.4  22.2  16.7  2.8  38.9 | |  |  |  |  |
| 1. *glucocorticoid alone** | Strongly agree  Somewhat agree  Somewhat disagree  Strongly disagree  Don’t know | 0.0  16.2  10.8  0.0  73.0 | 0.0  30.6  25.0  11.1  33.3 | |  |  |  |  |
| 1. *HPA & ANS activated** | Strongly agree  Somewhat agree  Somewhat disagree  Strongly disagree  Don’t know | 2.7  13.5  8.1  0.0  75.7 | 8.3  19.4  25.0  11.1  36.1 | |  |  |  |  |
| 1. *HPA & ANS retrieval** | Strongly agree  Somewhat agree  Somewhat disagree  Strongly disagree  Don’t know | 5.4  8.1  0.0  0.0  86.5 | 13.9  30.6  8.3  2.8  44.4 | |  |  |  |  |

*Note.* * = statement presented only to expert sample.

**Table B**

| *Percentage of Expert Agreement About Court Reliability and Research Basis for Each Statement* | | | |
| --- | --- | --- | --- |
| **Statement** |  | **Responses from those who agreed with statement**  **(% agreement)** | **Responses from those who disagreed with statement**  **(% agreement)** |
| 1. High stress impairs   64 agreed, 7 disagreed | Court reliability | 64.1 | 28.6 |
|  | Research basis | 95.3 | 100.0 |
| 1. Police interview   57 agreed, 8 disagreed | Court reliability | 64.9 | 50.0 |
|  | Research basis | 87.7 | 87.5 |
| 1. Stress impairs retrieval   65 agreed, 4 disagreed | Court reliability | 63.0 | 50.0 |
|  | Research basis | 95.4 | 100.0 |
| 1. Stress enhances encoding   40 agreed, 30 disagreed | Court reliability | 57.5 | 40.0 |
|  | Research basis | 97.5 | 96.7 |
| 1. Children less affected   3 agreed, 47 disagreed | Court reliability | 100.0 | 42.6 |
|  | Research basis | 66.7 | 74.5 |
| 1. Detail type   58 agreed, 10 disagreed | Court reliability | 81.0 | 40.0 |
|  | Research basis | 100.0 | 90.0 |
| 1. Test type   47 agreed, 10 disagreed | Court reliability | 63.8 | 20.0 |
|  | Research basis | 87.2 | 70.0 |
| 1. Faces affected differently   21 agreed, 26 disagreed | Court reliability | 66.7 | 19.2 |
|  | Research basis | 90.5 | 69.2 |
| 1. Professionals less affected   12 agreed, 58 disagreed | Court reliability | 16.7 | 37.9 |
|  | Research basis | 58.3 | 74.1 |
| 1. Victims more affected   49 agreed, 14 disagreed | Court reliability | 51.0 | 21.4 |
|  | Research basis | 69.4 | 57.1 |
| 1. Repression   11 agreed, 53 disagreed | Court reliability | 54.5 | 47.2 |
|  | Research basis | 81.8 | 90.6 |
| 1. Violent events   20 agreed, 40 disagreed | Court reliability | 80.0 | 45.0 |
|  | Research basis | 85.0 | 77.5 |
| 1. Emotional better remembered   45 agreed, 15 disagreed | Court reliability | 73.3 | 40.0 |
|  | Research basis | 91.1 | 86.7 |
| 1. Moderate stress   48 agreed, 13 disagreed | Court reliability | 58.3 | 30.8 |
|  | Research basis | 97.9 | 76.9 |
| 1. Severe stress   54 agreed, 14 disagreed | Court reliability | 66.7 | 57.1 |
|  | Research basis | 92.6 | 78.6 |
| 1. Short crime   1 agreed, 67 disagreed | Court reliability | 0.0 | 47.8 |
|  | Research basis | 0.0 | 82.1 |
| 1. Abstractness   18 agreed, 32 disagreed | Court reliability | 62.1 | 37.5 |
|  | Research basis | 89.7 | 90.6 |
| 1. Immediate retrieval enhances   19 agreed, 29 disagreed | Court reliability | 57.9 | 44.8 |
|  | Research basis | 84.2 | 89.2 |
| 1. Retrieval timing   34 agreed, 22 disagreed | Court reliability | 55.9 | 36.4 |
|  | Research basis | 82.4 | 81.8 |
| 1. Misinformation protection   11 agreed, 41 disagreed | Court reliability | 36.4 | 29.3 |
|  | Research basis | 90.9 | 78.0 |
| 1. Older adults less affected   5 agreed, 33 disagreed | Court reliability | 40.0 | 33.3 |
|  | Research basis | 60.0 | 63.6 |
| 1. Primarily ANS activity   24 agreed, 26 disagreed | Court reliability | 54.2 | 30.8 |
|  | Research basis | 87.5 | 92.3 |
| 1. ANS facilitates   52 agreed, 6 disagreed | Court reliability | 71.2 | 33.3 |
|  | Research basis | 92.3 | 83.3 |
| 1. Rapid cortisol is beneficial   24 agreed, 5 disagreed | Court reliability | 50.0 | 20.0 |
|  | Research basis | 79.2 | 100.0 |
| 1. Slow cortisol is detrimental   15 agreed, 12 disagreed | Court reliability | 40.0 | 25.0 |
|  | Research basis | 86.7 | 100.0 |
| 1. Noradrenergic alone   23 agreed, 10 disagreed | Court reliability | 60.9 | 30.0 |
|  | Research basis | 95.7 | 100.0 |
| 1. Glucocorticoid alone   17 agreed, 17 disagreed | Court reliability | 47.1 | 23.5 |
|  | Research basis | 94.1 | 88.2 |
| 1. HPA & ANS activated   16 agreed, 16 disagreed | Court reliability | 56.3 | 18.8 |
|  | Research basis | 87.5 | 93.8 |
| 1. HPA & ANS retrieval   21 agreed, 4 disagreed | Court reliability | 61.9 | 50.0 |
|  | Research basis | 85.7 | 100.0 |

*Note.* Court reliability = percentage of this subset of participants who said the statement was reliable enough for psychologists to present in the courtroom. Research basis = percentage of this subset of participants who said their opinion was based on published, peer reviewed, and scientific research.

| **Table C**  *Percentage of Memory Experts who Think Most People Believe This Statement to be True as a Matter of Common Sense* | | | |
| --- | --- | --- | --- |
| **Statement** | **Agree** | **Disagree** | **Don’t know** |
| 1. High stress impairs | 39.7 | 39.7 | 20.5 |
| 1. Police interview | 39.7 | 26.0 | 34.2 |
| 1. Stress impairs retrieval | 42.5 | 24.7 | 32.9 |
| 1. Stress enhances encoding | 30.1 | 42.5 | 27.4 |
| 1. Children less affected | 6.8 | 45.2 | 47.9 |
| 1. Detail type | 24.7 | 41.1 | 34.2 |
| 1. Test type | 15.1 | 37.0 | 47.9 |
| 1. Faces affected differently | 13.7 | 35.6 | 50.7 |
| 1. Professionals less affected | 47.9 | 23.3 | 28.8 |
| 1. Victims more affected | 38.4 | 21.9 | 39.7 |
| 1. Repression | 49.3 | 26.0 | 24.7 |
| 1. Violent events | 17.8 | 42.5 | 39.7 |
| 1. Emotional better remembered | 34.2 | 21.9 | 43.8 |
| 1. Moderate stress | 13.7 | 38.4 | 47.9 |
| 1. Severe stress | 28.8 | 31.5 | 39.7 |
| 1. Short crime | 9.6 | 43.8 | 46.6 |
| 1. Abstractness | 4.1 | 50.7 | 45.2 |
| 1. Immediate retrieval enhances | 5.5 | 41.1 | 53.4 |
| 1. Retrieval timing | 15.1 | 30.1 | 54.8 |
| 1. Misinformation protection | 4.1 | 45.2 | 50.7 |
| 1. Older adults less affected | 11.9 | 35.6 | 53.4 |
| 1. Primarily ANS activity | 2.7 | 33.6 | 61.6 |
| 1. ANS facilitates | 19.2 | 41.1 | 39.7 |
| 1. Rapid cortisol is beneficial | 2.7 | 45.2 | 52.1 |
| 1. Slow cortisol is detrimental | 1.4 | 45.2 | 53.4 |
| 1. Noradrenergic alone | 6.8 | 41.1 | 52.1 |
| 1. Glucocorticoid alone | 0.0 | 46.6 | 53.4 |
| 1. HPA & ANS activated | 4.1 | 45.2 | 50.7 |
| 1. HPA & ANS retrieval | 2.7 | 42.5 | 54.8 |

| **Table D**  *Inferential Statistics for 2 x 2 χ2 Tests Comparing Selections (Don’t Know versus Agree/Disagree) in Eyewitness and Fundamental Memory Experts (df = 1)* | | | | | | | | |
| --- | --- | --- | --- | --- | --- | --- | --- | --- |
| Statement | *χ^2^* | *p* | *Adjusted p* | | φ | φ φ φ |  |  |
| 1. High stress impairs* |  | > .999 | .105 | .002 | | | |  |
| 1. Police interview* |  | > .999 | .105 | .005 | | | |  |
| 1. Stress impairs retrieval* |  | .115 | .105 | .237 | | | |  |
| 1. Stress enhances encoding* |  | > .999 | .105 | .066 | | | |  |
| 1. Children less affected | 3.397 | .065 | .105 | .216 | | | |  |
| 1. Detail type* |  | .358 | .105 | .159 | | | |  |
| 1. Test type | 0.254 | .614 | .105 | .059 | | | |  |
| 1. Faces affected differently | 1.134 | .287 | .105 | .125 | | | |  |
| 1. Professionals less affected* |  | .115 | .105 | .210 | | | |  |
| 1. Victims more affected* |  | .046 | .105 | .245 | | | |  |
| 1. Repression* |  | .308 | .105 | .130 | | | |  |
| 1. Violent events | 7.885 | .005 | .105 | .329 | | | |  |
| 1. Emotional better remembered | 0.063 | .801 | .105 | .029 | | | |  |
| 1. Moderate stress | 0.336 | .562 | .105 | .068 | | | |  |
| 1. Severe stress* |  | .025 | .105 | .275 | | | |  |
| 1. Short crime* |  | .674 | .105 | .058 | | | |  |
| 1. Abstractness | 6.124 | .013 | .105 | .290 | | | |  |
| 1. Immediate retrieval enhances | 0.110 | .741 | .105 | .039 | | | |  |
| 1. Retrieval timing | 0.117 | .733 | .105 | .040 | | | |  |
| 1. **Misinformation protection** | **10.805** | **.001** | **.023** | **.385** | | | |  |
| 1. Older adults less affected | 0.349 | .555 | .105 | .069 | | | |  |
| 1. **Primarily ANS activity** | **10.216** | **.001** | **.031** | **.374** | | | |  |
| 1. **ANS facilitates** | **13.738** | **< .001** | **.005** | **.434** | | | |  |
| 1. **Rapid cortisol is beneficial** | **17.319** | **< .001** | **< .001** | **.487** | | | |  |
| 1. **Slow cortisol is detrimental** | **13.887** | **< .001** | **.005** | **.436** | | | |  |
| 1. Noradrenergic alone | 7.254 | .007 | .105 | .315 | | | |  |
| 1. **Glucocorticoid alone** | **11.522** | **.001** | **.017** | **.397** | | | |  |
| 1. **HPA & ANS activated** | **11.601** | **.001** | **.017** | **.399** | | | |  |
| 1. **HPA & ANS retrieval** | **14.322** | **< .001** | **.004** | **.443** | | | |  |

*Note. n* = 73. *Adjusted p* = Holm-Bonferroni adjustment for multiple comparisons. **Bold** = *adjusted p* significant at the .05 level. * = Fisher’s exact test instead of chi-square test (when expected cell sizes < 5).

| \| **Table E**  *Inferential Statistics for 2 x 2 χ2 Tests Comparing Selections (Don’t Know versus Agree/Disagree) in Experts and Laypeople (df = 1)* \| \| \| \| \| \| --- \| --- \| --- \| --- \| --- \| \| Statement \| *χ^2^* \| *p* \| *Adjusted p* \| φ \| \| 1. High stress impairs* \|  \| > .999 \| .220 \| < .001 \| \| 1. Police interview \| 1.832 \| .176 \| .220 \| .100 \| \| 1. Stress impairs retrieval \| 0.841 \| .359 \| .220 \| .068 \| \| 1. Stress enhances encoding \| 3.939 \| .047 \| .220 \| .147 \| \| 1. Children less affected \| 6.454 \| .011 \| .220 \| .188 \| \| 1. Detail type \| 5.505 \| .019 \| .220 \| .174 \| \| 1. Test type \| 1.581 \| .209 \| .220 \| .093 \| \| 1. Faces affected differently \| 2.964 \| .085 \| .220 \| .128 \| \| 1. Professionals less affected* \|  \| .537 \| .220 \| .050 \| \| 1. Victims more affected \| 1.385 \| .239 \| .220 \| .087 \| \| 1. Repression \| 1.285 \| .257 \| .220 \| .084 \| \| 1. Violent events \| 0.061 \| .805 \| .220 \| .018 \| \| 1. Emotional better remembered \| 0.009 \| .926 \| .220 \| .007 \| \| 1. Moderate stress \| 0.248 \| .619 \| .220 \| .037 \| \| 1. Severe stress \| 1.680 \| .195 \| .220 \| .096 \| \| 1. Short crime \| 5.505 \| .019 \| . .220 \| .174 \| \| 1. Abstractness \| 0.030 \| .861 \| .220 \| .013 \| \| 1. Immediate retrieval enhances \| 2.655 \| .103 \| .220 \| .121 \| \| 1. Retrieval timing \| 0.658 \| .417 \| .220 \| .060 \| \| 1. Misinformation protection \| 0.551 \| .458 \| .220 \| .055 \| \| 1. **Older adults less affected** \| **16.881** \| **< .001** \| **< .001** \| **.305** \|   *Note*. *N* = 182. *Adjusted p* = Holm-Bonferroni adjustment for multiple comparisons. **Bold** = *adjusted p* significant at the .05 level. * = Fisher’s exact test instead of chi-square test (when expected cell sizes < 5). |
| --- | --- | --- | --- | --- | --- | --- | --- | --- | --- | --- | --- | --- | --- | --- | --- | --- | --- | --- | --- | --- | --- | --- | --- | --- | --- | --- | --- | --- | --- | --- | --- | --- | --- | --- | --- | --- | --- | --- | --- | --- | --- | --- | --- | --- | --- | --- | --- | --- | --- | --- | --- | --- | --- | --- | --- | --- | --- | --- | --- | --- | --- | --- | --- | --- | --- | --- | --- | --- | --- | --- | --- | --- | --- | --- | --- | --- | --- | --- | --- | --- | --- | --- | --- | --- | --- | --- | --- | --- | --- | --- | --- | --- | --- | --- | --- | --- | --- | --- | --- | --- | --- | --- | --- | --- | --- | --- | --- | --- | --- | --- | --- | --- | --- | --- | --- |
